# Electronic health records identify timely trends in childhood mental health conditions

**DOI:** 10.1186/s13034-023-00650-7

**Published:** 2023-09-14

**Authors:** Josephine Elia, Kathleen Pajer, Raghuram Prasad, Andres Pumariega, Mitchell Maltenfort, Levon Utidjian, Elizabeth Shenkman, Kelly Kelleher, Suchitra Rao, Peter A. Margolis, Dimitri A. Christakis, Antonio Y. Hardan, Rachel Ballard, Christopher B. Forrest

**Affiliations:** 1 Department of Pediatrics, Nemours Children’s Health Delaware, Sydney Kimmel School of Medicine, Philadelphia, PA US; 2 Department of Psychiatry, Faculty of Medicine, University of Ottawa, Children’s Hospital of Eastern Ontario, Ottawa, ON Canada; 3 Department of Child and Adolescent Psychiatry, Children’s Hospital of Philadelphia, Perelman School of Medicine, the University of Pennsylvania, Philadelphia, PA US; 4 Department of Psychiatry, University of Florida College of Medicine, University of Florida Health, Gainesville, FL US; 5 Department of Biomedical and Health Informatics, Children’s Hospital of Philadelphia, Philadelphia, PA US; 6 Department of Pediatrics, Children’s Hospital of Philadelphia, Perelman School of Medicine, University of Pennsylvania, Philadelphia, US; 7 Department of Health Outcomes and Biomedical Informatics, University of Florida College of Medicine, Gainesville, US; 8 The Research Institute, Nationwide Children’s Hospital, Department of Pediatrics, The Ohio State University College of Medicine, Ohio, US; 9 Department of Pediatrics, Children’s Hospital of Colorado, University of Colorado, Aurora, CO US; 10 James Anderson Center for Health Systems Excellence, Department of Pediatrics, Cincinnati Children’s Hospital Medical Center, University of Cincinnati, Cincinnati, OH US; 11 Center for Child Health, Behavior and Development, Department of Pediatrics, Seattle Children’s Hospital, University of Washington, Seattle, Washington, US; 12 Department of Psychiatry and Behavioral Sciences, Stanford University, Palo Alto, CA US; 13 Department of Psychiatry and Behavioral Sciences and Pediatrics, Ann & Robert H. Lurie Children’s Hospital, Chicago, IL US; 14 Applied Clinical Research Center, Children’s Hospital of Philadelphia, Department of Healthcare Management, Perelman School of Medicine, the University of Pennsylvania, Philadelphia, US

**Keywords:** Electronic Health Records, EHR-based typology, *ICD-CM*, Pediatric Mental Health Disorders, Demographic risks, Covid-19, Surveillance, Standalone symptoms, Adverse childhood experience

## Abstract

**Background:**

Electronic health records (EHRs) data provide an opportunity to collect patient information rapidly, efficiently and at scale. National collaborative research networks, such as PEDSnet, aggregate EHRs data across institutions, enabling rapid identification of pediatric disease cohorts and generating new knowledge for medical conditions. To date, aggregation of EHR data has had limited applications in advancing our understanding of mental health (MH) conditions, in part due to the limited research in clinical informatics, necessary for the translation of EHR data to child mental health research.

**Methods:**

In this cohort study, a comprehensive EHR-based typology was developed by an interdisciplinary team, with expertise in informatics and child and adolescent psychiatry, to query aggregated, standardized EHR data for the full spectrum of MH conditions (disorders/symptoms and exposure to adverse childhood experiences (ACEs), across 13 years (2010–2023), from 9 PEDSnet centers. Patients with and without MH disorders/symptoms (without ACEs), were compared by age, gender, race/ethnicity, insurance, and chronic physical conditions. Patients with ACEs alone were compared with those that also had MH disorders/symptoms. Prevalence estimates for patients with 1^+^ disorder/symptoms and for specific disorders/symptoms and exposure to ACEs were calculated, as well as risk for developing MH disorder/symptoms.

**Results:**

The EHR study data set included 7,852,081 patients < 21 years of age, of which 52.1% were male. Of this group, 1,552,726 (19.8%), without exposure to ACEs, had a lifetime MH disorders/symptoms, 56.5% being male. Annual prevalence estimates of MH disorders/symptoms (without exposure to ACEs) rose from 10.6% to 2010 to 15.1% in 2023, a 44% relative increase, peaking to 15.4% in 2019, prior to the Covid-19 pandemic. MH categories with the largest increases between 2010 and 2023 were exposure to ACEs (1.7, 95% CI 1.6–1.8), anxiety disorders (2.8, 95% CI 2.8–2.9), eating/feeding disorders (2.1, 95% CI 2.1–2.2), gender dysphoria/sexual dysfunction (43.6, 95% CI 35.8–53.0), and intentional self-harm/suicidality (3.3, 95% CI 3.2–3.5). White youths had the highest rates in most categories, except for disruptive behavior disorders, elimination disorders, psychotic disorders, and standalone symptoms which Black youths had higher rates. Median age of detection was 8.1 years (IQR 3.5–13.5) with all standalone symptoms recorded earlier than the corresponding MH disorder categories.

**Conclusions:**

These results support EHRs’ capability in capturing the full spectrum of MH disorders/symptoms and exposure to ACEs, identifying the proportion of patients and groups at risk, and detecting trends throughout a 13-year period that included the Covid-19 pandemic. Standardized EHR data, which capture MH conditions is critical for health systems to examine past and current trends for future surveillance. Our publicly available EHR-mental health typology codes can be used in other studies to further advance research in this area.

**Supplementary Information:**

The online version contains supplementary material available at 10.1186/s13034-023-00650-7.

## Background

Electronic health record (EHR) data provide an opportunity to collect patient and treatment information rapidly, efficiently, and at scale [1, 2], rendering them critical for modern health systems. The establishment of national collaborative research networks such as PEDSnet (http://pedsnet.org/), where multi-institution clinical EHR data are transformed into a common research format that allows data sharing and analyses across health systems, has enabled rapid identification of pediatric disease cohorts [3–5] and generated new knowledge for pediatric medical conditions [3, 6].

To date, routine applications of clinical informatics in pediatric mental health care are limited to point of care tools, such as rating scales for suicide and depression [7]. Large scale applications include a few studies during the Covid-19 pandemic aimed to understand health trends and track the rise in mental health needs [8–10], as well as a few studies querying IBM Medicaid MarketScan EHR records [11, 12].

Our interdisciplinary team of experts in informatics and Child and Adolescent Psychiatry collaborated in assessing the utilization of available, aggregated, standardized EHR data in identifying and tracking the full diagnostic spectrum of MH disorders/symptoms and exposure to ACEs, over time, across multiple sites and identifying groups at risk by year and demographics. This study necessitated the development of an EHR-based pediatric MH condition typology that expanded on the Diagnostic and Statistical Manual of Mental Disorders (DSM) by including Intentional Self-Harm, Catatonia, Encephalopathies, Standalone Symptoms, Tic Disorders, added exposure to ACEs, and that took into account the 2015 U.S. healthcare transition to the International Classifications of Diseases (*ICD-10)* [13]. This involved assembling clinical codes representing single diagnoses and symptoms from the Systematized Nomenclature of Medical Clinical Terms (SNOMED CT) [14, 15] which were then cross-mapped to *ICD-9* [16] and *ICD-10* [17] and checked for omission, commissions and alignment with DSM-5 [18].

Leveraging clinical informatics to advance our understanding of childhood mental health is critical. While morbidity and mortality have been decreasing for most child and adolescent conditions [19], data on pediatric MH conditions [20] has demonstrated a disturbing increase. The Great Recession of 2008 [21] was associated with increased risk for MH conditions and this trend has continued [22]. A screening program [23] reported worsening mental health, particularly in youths, even before the additional adverse impact of the Covid-19 pandemic [24] that is expected to persist even as the pandemic declines [25]. The increasing magnitude of this ongoing mental health crisis has led U.S. health-organizations to declare a national emergency [26].

Our study aims to contribute to the limited research in clinical informatics methodology by focusing on the translation of EHR data to child mental health care [11, 12, 27–29]. After developing the MH condition typology, we then used it to characterize the multi-hospital populations as a test case. The full list of codes and their assignments is publicly available for others to use to further advance mental health research using this typology to translate EHR data (https://github.com/PEDSnet/MHCC).

## Methods

The study was approved by the Institutional Review Board at the Children’s Hospital of Philadelphia (IRB protocol #17-014012), exempt from consent and Health Insurance Portability and Accountability Act authorization, and used the PEDSnet Master Reliance Agreement process whereby participating institutions ceded IRB review to the Children’s Hospital of Philadelphia.

We used the Strobe cohort checklist (additional File 1) when writing our report [30].

### Aims

The primary objective of this study was to assess the translation of available EHR data by developing a MH condition typology and characterizing a multi-hospital population as a test case, identifying, and tracking the full spectrum of pediatric MH disorders/symptoms and exposure to ACEs over time, across sites, clinical settings and diagnostic categories and identifying groups at risk by year and demographics.

### Design

We used a retrospective cohort study design with existing standardized EHR data collected between 2010 and 2023 from 9 PEDSnet institutions. A newly developed EHR-based pediatric MH condition typology was developed that expanded on the DSM disorders by including Intentional Self-Harm, Catatonia, Encephalopathies, Standalone Symptoms, Tic Disorders and added exposure to ACEs. Patients with and without MH disorders/symptoms (without ACEs), were compared by age, gender, race/ethnicity, insurance, and chronic physical conditions. Patients with ACEs alone were compared with those that also had MH disorders/symptoms. Prevalence estimates of patients with 1^+^ disorder/symptoms, specific disorder/symptoms and exposure to ACEs were calculated, as well as risk for developing a mental health disorder.

### Setting/Data source

The study used EHR data from nine health systems that participate in PEDSnet, a national clinical research network (https://pedsnet.org) that is part of PCORnet (The National Patient Centered Research Network) [31]. Each quarter, each PEDSnet center extracts, transforms and loads (ETL process) EHR data into a reporting database where the data undergoes terminology harmonization using the Observational Medical Outcome Partnership (OMOP) common data model [32]. It is then submitted to the PEDSnet coordinating center at the Children’s Hospital of Philadelphia where data quality assessments are conducted on each release [33] and feedback is provided to the sites for improving the individual datasets for the next iteration. PEDSnet ETL conventions are publicly available (https://pedsnet.org/resources/reference/).

The raw EHR data containing structured data (clinical codes for problems) and unstructured data (free text) require the construction of “code sets” [34]. This process involves assembling a set of clinical codes that represents a single concept such as a diagnosis. Once constructed, these code sets are used to query and extract data from the EHR database. The development of the EHR-based pediatric mental health typology, used in this study, started by assembling a set of clinical codes from the Systematized Nomenclature of Medicine Clinical Terms (SNOMED CT) [14, 35].

SNOMED CT, is a systematically organized computer processable collection of medical terms linked to codes, synonyms and definitions used in clinical documentation, and it provides a standard approach to indexing, storing and retrieving medical data across specialties and sites of care [14, 35]. We then used SNOMED CT to cross-map to the International Statistical Classification of Diseases and Related Problems (*ICD*) code set, maintained by the World Health Organization [36]. This is the most widely used statistical classification system for diseases in the world and is used as a diagnostic research tool for epidemiology and health management [36]. More specifically, we used SNOMED CT to cross-map to The International Classification of Diseases, Clinical Modification (*ICD-CM*), an adaption created by the U.S. National Center for Health Statistics, updated annually, and used in assigning diagnostic and procedure codes associated with inpatient, outpatient and physician utilization in the U.S [37]. Both SNOMED CT and *ICD* use standardized definitions and form a common medical language used within the electronic health record (EHR) systems [17]. SNOMED CT enables information input into an EHR system during the course of patient care, while ICD facilitates information retrieval, or output, for secondary data purposes [17].

Some important considerations for our study included the *ICD* code revisions. In 2015 US healthcare transition to the current International Classification of Diseases, Tenth Revision, Clinical Modifications (*ICD-10-CM*) coding [13]. This reclassified child mental health disorders were under new psychiatric diagnostic groups and diagnosis codes for individual disorders, that differed from the previous classifications (*ICD-9-CM*) [16]. Another consideration was the differences in granularity, emphasis and organizing principles between SNOMED CT and *ICD-10-CM*, where it is not always possible to have a one-to-one map between a SNOMED CT concept and an *ICD-10-CM* code.

A team of six clinicians (four child and adolescent psychiatrics and two general pediatricians with expertise in clinical informatics) participated in the development of an EHR-based typology. The final typology comprised of 16 MH condition categories (e.g., anxiety disorder) and 49 clusters pertaining to the categories (e.g., 4 clusters including Anxiety Disorders, Obsessive Compulsive, Somatoform and Stress Disorders). Another category focusing on exposure to ACEs, consisted of 4 clusters (e.g., emotional, physical and sexual, abuse and neglect). (Additional File 2 for the EHR-based pediatric MH condition typology). The number of SNOMED CT diagnostic terms per condition category ranged from 3 to 1185, while the number of clusters per category ranged from 1 to 5 (see Additional Files 3–8 for the Typology Development). Essentially, the EHR based typology we used to query the EHR database attempted to capture the full spectrum of pediatric MH disorders covered in the DSM-5 [18] and also include additional conditions such as Intentional Self-Harm, Catatonia, Encephalopathies, Standalone Symptoms, Tic Disorders and exposure to ACEs. The full list of codes and their assignments is publicly available (see https://github.com/PEDSnet/MHCC).

Institutions participating in this study included: Children’s Hospital of Colorado; Children’s Hospital of Philadelphia; Cincinnati Children’s Hospital Medical Center; Ann & Robert H. Lurie Children’s Hospital of Chicago; Nationwide Children’s Hospital; Nemours Children’s Health System (both Delaware and Florida health systems); Seattle Children’s Hospital; and Stanford Medicine Children’s Health. We used the PEDSnet database version 4.4 for this study.

### Sample

The sample included patients with one or more physician face-to-face encounters provided in outpatient, inpatient, and emergency department (ED) settings, from nine of the US’s largest health systems, participating in PEDSnet. Patients 21 years of age or older were excluded.

The study period was January 1, 2010, through March 31, 2023. Data were extracted for analysis in July 2023.

Covariates include participating institution, services (inpatient, outpatient, ED), timing of physician visits in calendar year and duration of follow-up (computed as the date of the last visit minus the date of the first visit), number of chronic physical conditions, and MH disorders/symptoms and exposure to ACEs. Patient demographic data included age, sex assigned at birth, insurance type, and race and ethnicity. Race and ethnicity classifications (Hispanic; if non-Hispanic then classify as white, Black, Asian/Pacific Islander, Other) were determined by self-identification.

### Statistical analysis

We contrasted patients with and without MH disorders/symptoms and with and without exposure to ACEs on age at first visit, duration of follow-up, sex assigned at birth, race/ethnicity, payer, and institution. We also used a modified Pediatric Medical Comorbidity Algorithm [38] to identify patients with chronic physical health conditions. To do this, we dropped mental health disorders from the list of chronic disease body systems and tallied the count of distinct body systems per patient [39].

Because sample sizes were large, we used the standardized difference statistic to identify meaningful differences in the two distributions. The standardized difference for proportions with > 2 categories is calculated from a normalized distance matrix describing differences between category counts. These calculations were implemented using the R *tableone* package [40]. We considered a difference of > 0.20 to be a meaningful effect size.

For each MH condition category (MH disorder/symptoms and ACE) and its member clusters, presented in (additional File 2), we computed the median age and interquartile range (IQR) when the patient received their first code for that condition and the proportion of patients who were male. Lastly, we estimated the prevalence of MH disorders/symptoms (with and without exposure to ACEs) in the full sample using all available data.

Annual calendar-year prevalence estimates were calculated from the denominator of the count of all patients younger than 21 years old having a visit during the calendar year and a numerator of the count of distinct patients with at least one visit for a MH condition (MH disorders/symptoms, ACEs). Prevalence estimates were calculated for January 2011 through March 2023 based on a one-year lookback (e.g., January 2010, etc.). We plotted annual rolling percentages of patients with a MH condition (MH disorders/symptoms, ACEs), each calendar month using a 12-month look-back and stratifying by sex and race ethnicity sub-groups, condition category, site, and service. As the database began in 2009, our estimates of MH visit rates per year showed discontinuities in that first year. To avoid artefacts distorting the results, we decided to drop the 2009 data and focus on the years 2010 and after.

To identify risk factors for any type of MH disorder/symptoms (with and without exposure to ACEs), we fit a Poisson regression with offset (denominator) of number of patients meeting the criteria, predicting the count of patients in the denominator with at least one MH disorder/symptom. A quasi-Poisson model was used to allow for over-dispersion in which the observed variance is larger than that assumed in the standard model. The model returned rate ratios that show how the baseline rate is affected by covariates. All analyses were performed in R 4.3 (R Core Team 2021) [41].

## Results

The entire PEDSnet overall population comprised 9,107,649 patients with one or more physician visits. As shown in Table 1, there were 7,852,081 patients younger than 21 years of age, from January 1, 2010, through March 31, 2023, and this comprised our study sample. A total of 1,552,726/7,852,081 (19.8%) of patients presented with a MH disorder/symptom (without ACEs) over the thirteen years under study, with varying rates of 6.1–19.5% among the nine participating sites. There was no difference between the patients with and without a MH disorder/symptoms (without exposure to ACEs), with regards to age, race and ethnicity, or insurance type while those with MH disorders/symptoms had a higher proportion of males, longer periods of follow-up (defined by number of years during which time additional visits for any condition occurred) compared with those without disorders (5.1 versus 2.4 years), even though mean age at entrance into the database was similar (6.5 vs. 6.4 years-old). Patients with MH disorders/ symptoms were also twice as likely to also be diagnosed with a chronic physical condition than those without (59.0% vs. 29.4%) at first visit and had a higher number of body systems involved (1.4 vs. 0.4).

**Table 1:** Study sample characteristics by presence of a mental health disorders/symptoms (dis/sx) with and without adverse childhood experiences (ACEs) and absence of all mental health conditions

	Overall Study >age 21	MH dis/sx. No ACE	No MH dis/sx. No ACE	SMD	MH dis/sx. + ACE	No MH dis/sx. +ACE	SMD	SMD (average)
**Sample Size** (n)	7,852,081 **(100%)**	1,552,726 **(19.8%)**	6,219,285 **(79.2%)**		46,277 **(0.59%)**	33,793 **(0.43%)**		
**Age at first visit, y**, mean/(SD)	6.4 (5.8)	6.5 (5.6)	6.4 (5.9)	0.006	6.3 (5.4)	4.3 (4.9)	0.388	0.213
**Age at first MH visit, y**, mean/(SD)	8.5(5.5)	8.6 (5.5)	NA	NA	8.5 (5.3)	NA	NA	NA
**Follow-up duration, y**, mean (SD)	3.0 (3.6)	5.1 (4.0)	2.4 (3.2)	0.731	7.0 (4.1)	4.1 (3.9)	0.725	0.645
**Sex**				1.07			0.067	0.264
Female	3,759,392 **(47.9%)**	674,607 **(43.4%)**	3,034,232 **(48.8%)**		28,632 **(61.9%)**	21,921 **(64.9%)**		
Male	4,091,978 **(52.1%)**	878,010 **(56.5%)**	3,184,466 **(51.2%)**		17,643 **(38.1%)**	11,859 **(35.1%)**		
**Race and Ethnicity**				0.155			0.118	0.297
Non-Hispanic White	4,020,014 **(51.2%)**	835,851 **(53.8%)**	3,146,217 **(50.6%)**		22,493 **(48.6%)**	15,453 **(45.7%)**		
Non-Hispanic Black	1,074,630 **(13.7%)**	247,805 **(16.0%)**	804,519 **(12.9%)**		13,269 **(28.7%)**	9,037 **(26.7%)**		
Hispanic	1,243,842 **(15.8%)**	231,991 **(14.9%)**	1,001,259 **(16.1%)**		5,428 **(11.7%)**	5,164 **(15.3%)**		
Asian/Pacific	351,196 **(4.5%)**	55,262 **(3.6%)**	294,947 **(4.7%)**		552 **(1.2%)**	435 **(1.3%)**		
Other/ Unknown	1,162,399 **(14.8%)**	181,817 **(11.7%)**	972,343 **(15.6%)**		4,535 **(9.8%)**	3,704 **(11%)**		
**Year of first visit**				0.387			0.349	0.387
2010	1,369,494 **(17.4%)**	395,255 **(25.5%)**	951,877 **(15.3%)**		16,715 **(36.1%)**	5,647 **(16.7%)**		
2011	638,125 **(8.1%)**	137,324 **(8.8%)**	492,450 **(7.9%)**		5,260 **(11.4%)**	3,091 **(9.1%)**		
2012	534,633 **(6.8%)**	110,516 **(7.1%)**	417,775 **(6.7%)**		3,839 **(8.3%)**	2,503 **(7.4%)**		
2013	509,932 **(6.5%)**	105,812 **(6.8%)**	398,509 **(6.4%)**		3,335 **(7.2%)**	2,276 **(6.7%)**		
2014	617,351 **(7.9%)**	127,413 **(8.2%)**	484,523 **(7.8%)**		3,099 **(6.7%)**	2,316 **(6.9%)**		
2015	584,292 **(7.4%)**	113,236 **(7.3%)**	465,769 **(7.5%)**		2,846 **(6.1%)**	2,441 **(7.2%)**		
2016	575,099 **(7.3%)**	108,133 **(7.0%)**	461,876 **(7.4%)**		2,512 **(5.4%)**	2,578 **(7.6%)**		
2017	557,137 **(7.1%)**	103,608 **(6.7%)**	448,866 **(7.2%)**		2,282 **(4.9%)**	2,581 **(7.6%)**		
2018	547,859 **(7.0%)**	97,290 **(6.3%)**	446,173 **(7.2%)**		1,931 **(4.2%)**	2,465 **(7.3%)**		
2019	501,325 **(6.4%)**	83,364 **(5.4%)**	414,133 **(6.7%)**		1,547 **(3.3%)**	2,281 **(6.7%)**		
2020	389,391 **(5.0%)**	60,941 **(3.9%)**	325,308 **(5.2%)**		1,214 **(2.6%)**	1,928 **(5.7%)**		
2021	475,557 **(6.1%)**	61,416 **(4.0%)**	411,161 **(6.6%)**		1,002 **(2.2%)**	1,978 **(5.9%)**		
2022	469,455 **(6.0%)**	42,587 **(2.7%)**	424,746 **(6.8%)**		624 **(1.3%)**	1,498 **(4.4%)**		
2023	82,431 **(1.0%)**	5,831 **(0.4%)**	76,319 **(1.2%)**		71 **(0.2%)**	210 **(0.6%)**		
**Insurance type at first visit**				0.165			0.161	0.511
Private/ Commercial	3,548,463 **(45.2%)**	607,246 **(39.1%)**	2,930,195 **(47.1%)**		5,676 **(12.3%)**	5,346 **(15.8%)**		
Public	2,879,351 **(36.7%)**	618,049 **(39.8%)**	2,215,948 **(35.6%)**		25,609 **(55.3%)**	19,745 **(58.4%)**		
Other/ Unknown	1,424,267 (**18.1%)**	327,431 **(21.1%)**	1,073,142 **(17.3%)**		14,992 **(32.4%)**	8,702 **(25.8%)**		
**Number of body systems**, (mean/SD)	0.6 (1.2)	1.4 (1.9)	0.4 (0.8)	0.638	1.5 (2.0)	0.4 (0.8)	0.072	0.463
**Number of chronic physical health condition body systems** ^1^				0.727			0.813	0.542
0	**64.5%**	**41.0%**	**70.6%**		**37.3%**	**71.2%**		
1	**23.1%**	**28.3%**	**21.8%**		**27.4%**	**19.9%**		
2	**7%**	**14.2%**	**5.2%**		**15.9%**	**6.0%**		
3+	**5.3%**	**16.5%**	**2.4%**		**19.5%**	**3%**		
**PEDSnet Health Sites**, n/ (%)				0.282			0.358	0.625
**A**	1,409,799 **(18%)**	302,523 **(19.5%)**	1,098,750 **(17.7%)**		4,393 **(9.5%)**	4133 **(12.2%)**		
**B**	534,446 **6.8%**	100,103 **(6.4%)**	430,555 **(6.9%)**		2,484 **(5.4%)**	1,304 **(3.9%)**		
**C**	1,131,781 **(14.4%)**	209,940 **(13.5%)**	913,898 **(14.7%)**		4,282 **(9.3%)**	3,661 **(10.8)**		
**D**	981,695 **(12.5%)**	214,429 **(13.8%)**	745,523 **(12.0%)**		12,086 **(26.1%)**	9,657 **(28.6%)**		
**E**	906,216 **(11.5%)**	251,497 **(16.2%)**	632,325 **(10.2%)**		15,528 **(33.6%)**	6,866 **(20.3%)**		
**F**	534,189 **(6.8%)**	94,104 **(6.1%)**	439,468 **(7.1%)**		351 **(0.8%)**	266 **(0.8%)**		
**G**	739,150 **(9.4%)**	98,932 **(6.4%)**	635,029 **(10.2%)**		2,067 **(4.5%)**	3,122 **(9.2%)**		
**H**	636,377 **(8.1%)**	147,263 **(9.5%)**	480,718 **(7.7%)**		4,241 **(9.2%)**	4,155 **(12.3%)**		
**I**	978,428 (**12.5%)**	133,935 **(8.6%)**	843,019 **(13.6%)**		845 **(1.8%)**	629 **(1.9%)**		

***Note***: *Standardized Difference****(SMD****) = the statistic used to identify meaningful differences in two distributions (e.g., patients with mental health disorders/symptoms and no ACEs versus patients with no MH disorders/symptoms and no ACEs; patients with ACEs alone versus patients with ACEs + MH disorders/symptoms). SMD average is an average of all possible pairwise SMD’s. A difference of > 0.20 is a meaningful effect size*

*N = Sample size; y- Year; SD = Standard Deviation*

*Race and Ethnicity = self-identified*

^*1*^ *Using the Pediatric Medical Complexity Algorithm(39), diagnosis terms were assigned to body systems (e.g., cardiac, endocrine, etc.), excluding the mental health condition category*

*PEDSnet Health Sites = anonymized A through I*

*MH = Mental Health*

*dis = disorder(s)*

*sx = symptom(s)*

*ACEs = Adverse Childhood Experience(s)*

Compared to patients without ACEs, those with ACEs had a higher proportion of females, Blacks and higher rates of public insurance.

Patients exposed to ACEs that also had one or more MH disorders/symptoms differed from the patients with ACEs alone in that they were older (6.3 vs. 4.3 years), had longer follow-up (7 vs. 4.1 years) and were three times as likely to be diagnosed with a chronic physical condition (62.8% vs. 26.2%), but did not differ with regards to sex or race/ethnicity or insurance type.

**Fig. 1:**
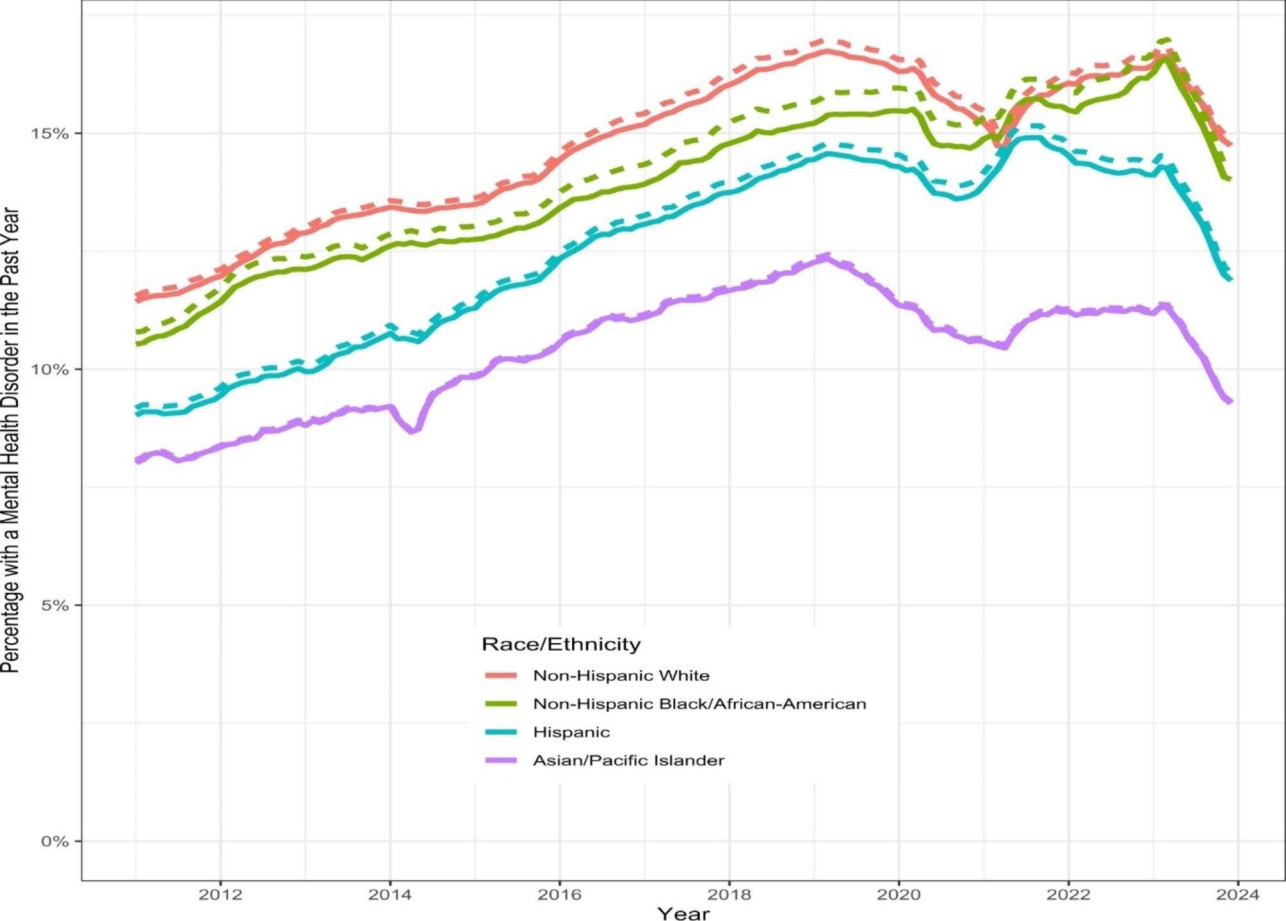
Annual rolling percentages of patients < 21 years-old with a mental health disorder/ symptom (dashed lines include ACE exposures), stratified by race and ethnicity, 2011 to 2023

The annual prevalence estimates of patients with one or more MH disorders/symptoms (without exposure to ACEs) within a calendar year rose from 10.6% to 2010 to 15.1% in 2023 with variations across sites (see Additional File 9, Fig. 1). Slightly higher rates of 10.8–15.4% when ACE exposures were included. As shown in Fig. 1, stratification by race and ethnicity demonstrated increases over time for all sub-groups up to 2019, slight decreases for all groups except Black youths until the onset of Covid-19 in 2020 that persisted until 2021 with subsequent increases, maintained throughout 2022 in whites and Blacks, plateauing in the Asian/Pacific group and decreasing in Hispanics. White youths starting and maintaining the highest prevalence estimates and patients of Asian/Pacific Islander backgrounds having the lowest estimates for disorder categories/symptoms.

Of the sixteen MH condition categories, nine showed a monotonic and progressive rise in the annual rolling rates across the study period and across sites (see Additional File 10, Fig. 2). The difference of 2023 versus 2010 proportion rate ratio for each of these 9 condition categories were: adverse childhood experiences (1.7, 95% CI 1.6–1.8); anxiety disorders (2.8, 95% CI 2.8–2.9); eating/feeding disorders (2.1, 95% CI 2.1–2.2); gender dysphoria/sexual dysfunction (43.6, 95% CI 35.8–53.0); intentional self-harm/suicidality (3.3, 95% CI 3.2–3.5); mood disorders (1.7, 95% CI 1.7–1.8); neurodevelopmental disorders (1.4, 95% CI 1.4–1.4); sleep-wake disorders (1.8, 95% CI 1.8–1.9); and standalone symptoms (1.8, 95% CI 1.8–1.8).

**Fig. 2:**
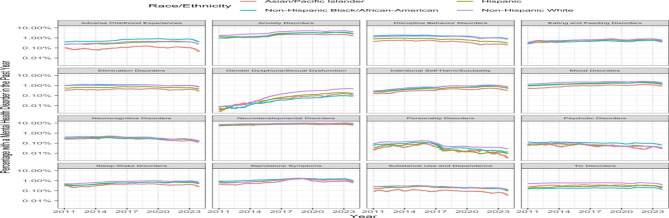
Annual rolling percentages of patients < 21 years-old with a mental health condition category (disorder/symptoms, exposure to ACEs) stratified by race and ethnicity, 2011 to 2023

As shown in Fig. 2, white youths were shown to have the highest prevalence estimates in most categories while Black youths had higher estimates of exposure to ACEs, disruptive behavior disorders, psychotic disorders, and standalone symptoms. Asian/Pacific Islanders tended towards the lowest estimates except for eating and feeding disorders, gender dysphoria and tic disorders with Blacks having the lowest estimates for gender dysphoria and tic disorders.

The smallest variation between the different ethnic and racial groups was noted in the neurodevelopmental and neurocognitive categories. Despite variations in proportions between the different ethnic and racial groups, curves were generally consistent across races except for gender dysphoria where the increase was sharp for white youths. In the first several months of 2020, coinciding with the start of the Covid-19 pandemic quarantine that limited in-person visits, there was a decrease in prevalence estimates in all the 16 categories except for intentional self-harm that plateaued for whites and Hispanics and gender dysphoria which increased for all groups except Asian Pacific youths.

**Fig. 3:**
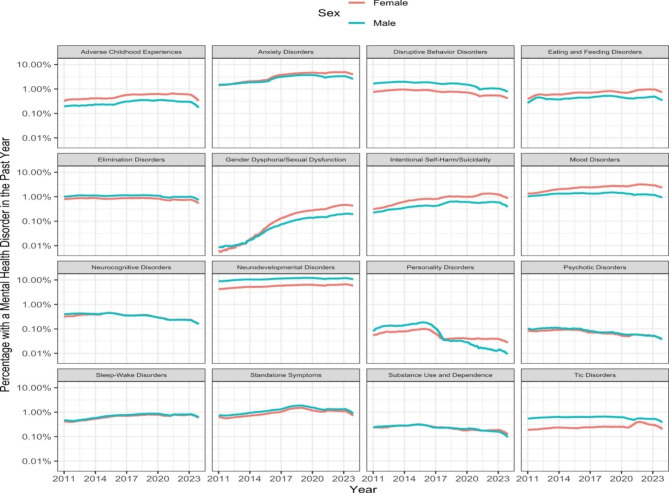
Annual rolling percentages of patients < 21 years-old with distinct categories of mental health conditions (disorders/symptoms, exposure to ACEs) by sex, 2011 to 2023

Males started with higher prevalence estimates of MH disorders/symptoms than females and this pattern continued throughout the study period. Diagnostic categories with the highest proportion estimates of male patients were tic disorders (70.0%), disruptive behavior disorders (67.5%), and neurodevelopmental disorders (65.9%). Clusters with highest proportion estimates of males were Autism (77%), paraphilia (77%), Conduct (69%), Impulse control (68%), Oppositional defiant (68%), Communication/motor (66%) and Academic developmental disorders (60%), as well as ADHD (69%), Intellectual disability (63%), Encopresis (63%), and standalone Anger/aggressive 66%) and Attention symptom (60%). The categories with the highest proportion estimates of female patients were exposure to adverse childhood events (63.2%), gender dysphoria/sexual dysfunction (67.1%), mood disorders (63.0%), and intentional self-harm/suicidality (63.0%). The clusters with the highest proportion estimates of females were sexual abuse (78.4%), physical abuse (58.2%), gender dysphoria (68.5%), parasuicidality (67%), suicidality (63.7%), avoidant/restrictive food intake disorder (61.6%), somatoform disorder (62%), major depression (67,1%) and minor depression (62.5%) (see Table 2; Fig. 3).

As shown in Table 2, the median age of clinical detection of any MH condition (disorders/ symptoms or exposure to ACEs) was 8.1 years (IQR 13.5–13.5) with overall prevalence estimates of 20.4% for those without ACE exposures. Substance use and dependence had the oldest age of detection among categories (median age 16.4 years; IQR 14.4–17.6). Opioid-related substance use disorder had a median age of first diagnosis of just 4.4 years, but an IQR of 0.3–16.8, reflecting inclusion of children with neonatal abstinence syndrome and youth with opioid use disorder in the same category. The youngest median age of first category was for neurodevelopmental disorders (6.3 years; IQR 2.5–11.0). The standalone symptoms were all recorded earlier than the corresponding diagnostic categories (see Table 2).

**Table 2:** Mental Health (MH) condition categories (disorders/symptoms, exposure to ACEs, by age at first diagnosis, sex, and prevalence estimates

Category	Cluster	Age of first diagnosis, median (IQR)	% Male	Prevalence per 1,000 patients
**Any mental health disorder/symptom + ACE**		8.1 (3.4–13.4)	55.6	207.9
**Any mental health disorder/symptom**		8.1 (3.5–13.5)	56.0	203.6
Adverse childhood experiences	Overall (entire cluster)	8.4 (3.8–13.6)	36.8	10.2
	Emotional abuse	11.8 (9.0-14.2)	48.1	0.6
	Neglect	6.0 (2.5–11.9)	48.8	0.8
	Physical abuse	8.6 (3.0–14.0)	41.8	6.5
	Sexual abuse	9.7 (5.2–14.0)	21.6	4.5
Anxiety disorders	Overall (entire cluster)	12.4 (8.0-15.5)	45.7	58.1
	Anxiety disorder	12.8 (8.2–15.8)	44.3	38.6
	Obsessive-compulsive disorder	12.9 (9.6–15.6)	51.0	4.2
	Somatoform disorder	13.6 (9.9–16.1)	38.4	5.8
	Stress disorder	12.3 (8.1–15.4)	45.8	20.9
Disruptive behavior disorders	Overall (entire cluster)	8.8 (5.7–12.8)	67.5	19.9
	Conduct disorder	8.3 (5.0-12.6)	68.8	7.3
	Impulse control	8.5 (5.5–12.5)	68.2	12.0
	Oppositional defiant disorder	10.5 (7.7–13.6)	68.0	6.9
Eating & feeding disorders	Overall (entire cluster)	10.2 (3.4–15.3)	41.2	13.1
	Avoidant/restrictive food intake disorder	12.1 (4.3–15.7)	38.4	10.8
	Other eating & feeding disorder	4.6 (2.3–9.4)	53.7	2.9
Elimination disorders	Overall (entire cluster)	8.0 (6.0–10.5)	57.9	19.2
	Encopresis	7.3 (5.4–9.7)	63.0	5.6
	Enuresis	8.2 (6.2–10.8)	56.0	14.5
Gender dysphoria/sexual dysfunction	Overall (entire cluster)	15.3 (13.5–16.9)	32.9	2.1
	Gender Dysphoria	15.3 (13.5–16.9)	31.5	2.0
	Paraphilia	15.0 (13.0-16.7)	77.3	0.0
	Sexual Dysfunction	15.8 (13.3–18.0)	51.1	0.1
Intentional self-harm/suicidality	Overall (entire cluster)	14.5 (12.5–16.2)	37.4	15.9
	Parasuicidality	14.2 (11.8–15.9)	33.0	4.4
	Suicidality	14.7 (12.9–16.3)	36.3	13.9
Mood disorders	Overall (entire cluster)	15.0 (13.0-16.6)	37.1	34.5
	Bipolar Disorder	14.3 (10.5–16.6)	43.9	1.2
	Major Depression	15.3 (13.6–16.8)	32.9	17.6
	Minor Depression	15.0 (12.9–16.6)	37.5	27.2
Neurocognitive disorders	Overall (entire cluster)	12.5 (6.3–15.8)	52.3	7.6
	Catatonia	15.0 (10.4–16.9)	52.1	0.1
	Delirium	12.9 (6.6–16.0)	51.8	6.7
	Encephalopathy	9.6 (4.7–14.1)	56.0	0.9
Neurodevelopmental disorders	Overall (entire cluster)	6.3 (2.5–11.0)	65.9	112.9
	Academic developmental disorder	9.7 (7.7–12.7)	59.9	12.2
	Attention deficit disorder	10.3 (7.6–13.7)	68.8	44.3
	Autism spectrum disorder	6.8 (3.8–11.3)	77.1	22.5
	Communication/motor disorder	3.0 (1.8–5.4)	66.3	54.0
	Intellectual disability disorder	4.5 (2.0-9.7)	62.8	29.7
Personality disorders	Personality disorder (total cluster)	12.0 (7.6–15.6)	59.4	1.6
Psychotic disorders	Overall (entire cluster)	15.1 (12.4–16.9)	54.9	1.7
	Psychotic disorder	15.0 (12.2–16.8)	55.1	1.4
	Schizoaffective disorder	16.4 (14.8–17.6)	53.9	0.1
	Schizophrenia	16.1 (13.7–17.5)	58.3	0.3
Sleep-wake disorders	Overall (entire cluster)	10.0 (5.1–14.9)	52.9	15.1
	Hypersomnia	12.8 (8.1–15.9)	51.1	2.0
	Insomnia	11.4 (6.0-15.6)	50.8	9.8
	Parasomnias	7.2 (3.8–11.7)	57.1	5.4
Standalone symptoms	Overall (entire cluster)	8.2 (3.0–13.3)	56.1	28.3
	Anger/aggressive symptoms	9.4 (4.7–13.6)	66.0	7.8
	Anxiety symptoms	4.7 (0.8–12.1)	49.7	11.3
	Attention symptoms	9.0 (6.5–13.1)	60.2	7.5
	Depressive symptoms	8.1 (1.4–14.1)	44.5	1.6
	Hallucinations	13.3 (9.9–15.7)	47.7	1.8
Substance use & dependence	Overall (entire cluster)	16.4 (14.7–17.6)	52.3	5.9
	Alcohol substance use disorder	16.6 (15.4–17.6)	48.5	1.2
	Opioid-related substance use disorder	4.4 (0.3–16.8)	54.6	0.8
	Other substance use disorder	15.9 (12.2–17.3)	54.9	2.1
	Cannabis use disorder	16.6 (15.5–17.6)	54.0	2.3
	Tobacco use disorder	17.1 (15.9–18.1)	49.1	1.7
Tic Disorders	Tic Disorders (total cluster)	9.2 (6.5–12.2)	69.5	8.1

***Note***: *IQR = Interquartile ranges*

Annual prevalence estimates of patients with one or more MH disorder/symptoms and exposure to ACEs was slightly higher (10.8 to 15.4 vs. 10.6 to 15.1%) rose across all sites, and services (outpatient, inpatient, ED) with some variations among the 9 participating sites. Trajectories over time show increasing rates, peaking up to 15.7% and 15.4% in those with and without ACE exposure respectively, prior to the Covid-19 pandemic and a decrease at all sites at the beginning of 2020, followed by reversals in 2021, and subsequent continued increases or plateauing at most sites (see Additional Files 9, Fig. 1 and File 10, Fig. 2).

The percentage of patients with a MH disorder/symptom utilizing the three service settings, within the context of all pediatric patients, increased approximately 5% in all services, with inpatient, outpatient, and ED rates at approximately 20%, 15% and 10% respectively, with some small variability across sites. A sharp increase in ED utilization and decreases in the other two services were noted at the beginning of 2020 with the onset of Covid-19, with reversals in those trends in 2021. Estimates for those with ACE exposures are slightly higher in the inpatient and ED but follow similar trajectories in all 3 services (see Additional Files 11 & 12, Figs. 3 and 4). While prevalence estimates of MH disorders/symptoms increased throughout the study period in each of the three services, the proportion of mental health patients utilizing each of the 3 services remained roughly the same, with outpatient, ED and inpatient, accounting for approximately 80%, 15% and 5% respectively (see Additional File 13, Fig. 5). The trajectories cluster together for inpatient, and with some variations across sites for outpatient and ED (see Additional File, 14 Fig. 6).

When all patients were considered, outpatient services had greater utilization for most categories, other than tic and elimination disorders, managed mostly in the inpatient service. The ED had higher rates for exposure to ACEs, intentional self-arm, mood disorders, psychotic disorders, and substance use (see Additional File, 15 Fig. 7). When only mental health patients were considered, the highest utilization occurred in the outpatient services, with the ED setting approximately at the same level of use for exposure to ACEs, psychotic, and substance use disorders, and the highest level of use for intentional self-harm/suicidality (see Additional File 16, Fig. 8).

As shown in Table 3, the risk of being identified with a MH disorder/symptom increased from 2010 to 2018 with subsequent decreases coinciding with Covid-19 pandemic, reversing in 2022. Adolescents, males, whites, and those with public insurance were at higher risk for experiencing a MH disorder/symptoms (see Table 3). Similar results were found with exposure to ACEs.

**Table 3:** Risk of a mental health (MH) disorder(s)/symptom without exposure to ACEs by study year and demographics

	MH Disorder/Symptoms Without ACE
Predictor	Rate ratio (95% CI
**Calendar year**	
2010	*Referent*
2011	1.05 (1.02–1.08)
2012	1.12 (1.09–1.15)
2013	1.17 (1.14–1.20)
2014	1.19 (1.16–1.21)
2015	1.27 (1.24–1.30)
2016	1.33 (1.30–1.36)
2017	1.40 (1.37–1.43)
2018	1.46 (1.42–1.49)
2019	1.43 (1.40–1.46)
2020	1.33 (1.30–1.36)
2021	1.39 (1.36–1.42)
2022	1.41 (1.38–1.44)
2023	1.28 (1.24–1.32)
**Age, years**	
0	0.22 (0.21–0.23)
1–3	0.66 (0.65–0.67)
4–6	0.81 (0.80–0.82)
7–9	0.98 (0.97–0.99)
10–12	*Referent*
13–15	1.08 (107 − 1.09)
16–20	1.16 (1.14–1.17)
**Sex**	
Female	*Referent*
Male	1.35(1.34–1.36)
**Race and ethnicity**	
White	*Referent*
Hispanic	0.77 (0.76–0.78)
Asian/Pacific Islander	0.74 (0.72–0.75)
Black/African American	0.83 (0.82–0.83)
Other/Unknown	0.80 (0.79–0.81)
**Insurance type**	
Private/Commercial	*Referent*
Public	1.50 (1.48–1.51)
Other/Unknown	1.48 (1.46–1.49)

***Note***: *CI = Confidence Interval*

## Discussion

In this study, standardized existing EHR PEDSnet data was queried, using our newly developed EHR-based typology, to estimate the prevalence of pediatric MH conditions (disorders/symptoms, exposure to ACEs), detect time trends over 13 years, across sites, clinical settings, and diagnostic categories, and identify risks by year and patient demographics.

Results from our study show that the overall prevalence estimates of children and youth ever diagnosed with a MH disorder/symptoms was 19.8%, slightly higher than that reported by Zima [11] (17.3%) and Doupnik [12] (18.8%) in studies that queried IBM Medicaid MarketScan EHR records. Rates in the cross-sectional US National Survey of Children’s Health (NSCH) based on parental/caregiver survey on health conditions (ever diagnosed; current) averaged at 16.5% with state-level prevalence for at least one mental health disorder ranging from 7.6 to 27.2% [42] and showing incremental increases ranging from 9.2%, 16.5% and 21.9% for 2003, 2016 [42] and 2017–2018 [43] respectively. The slightly higher rates from our study may be due to the greater EHR detail than present in administrative claims data, increased awareness and screenings of mental health disorders [44], the comprehensive typology that expanded on the DSM, including Intentional Self-Harm, Catatonia, Encephalopathies, Standalone symptoms and Tic Disorders and the large number of children with medical conditions [45] in the PEDSnet data. In the Ontario Child Health Study, where population level survey data was linked with health administrative data, 21.7% of children ages 4–17 were reported to have had a mental health related service that included not only physician visits, similar to our study, but also other mental health provider visits [46, 47].

Our data showed annual prevalence estimates of children with one or more MH disorder/symptoms (without ACE exposure) progressively increasing from 10.6% to 2010 to 15.1% in 2023. Lower estimates reported in a worldwide pooled meta-analysis (13.4%) [48], a US cross-sectional survey of 8–15 year old children diagnosed by a structured interview (12- month prevalence rates of 13.1% without and 11.3% with impairment) [49], and a US longitudinal community study of 9–16 year old (13%) [50], may reflect data from past years and limited age ranges. The time-period of our study was longer and included recent years.

Our data on patients with ACE exposures shows slightly higher prevalence estimates of ever having a MH condition compared to those without ACE exposures (20.4% vs. 19.8%) as well as higher annual estimates (10.8–15.4% vs. 10.6–15.1%). This preliminary data on exposure to ACEs in our study provides support in the ability of the EHR-based typology to extract data related to potential determinants of MH disorder/symptoms. Given that only a few adverse exposures (derived from clinical codes and not from ACE rating scales) were included in our typology, our results are underestimates. Future studies with expanded typology categories on MH determinants may allow a more comprehensive extraction of this critical data.

The median age of clinical detection for any MH disorder/symptoms in our study is 8.1 years (IQR 3.5–13.5). The earliest and latest age of detection were 6.5 (IQR 2.6–11.1) for neurodevelopmental disorders and 16.2 years (IQR 14.4–17.5) for substance use and dependence disorders. The anxiety disorders with a median age of onset at 12.4 (IQR 8-15.5) preceded the mood and psychotic disorders both with median age of onset at age 15. Interestingly, all standalone symptoms preceded the onset of their respective disorders suggesting that this category may be extremely important in identifying prodromal manifestations, critical in the development of preventive strategies. As would be expected, higher median ages of onset are reported for any disorder (18 years; IQR 11–34) and across all disorders, in a meta-analysis of worldwide epidemiological studies across the lifespan [51].

Which MH conditions may be contributing to the increase in prevalence estimates? In our study, nine of the sixteen categories showed a progressive rise across the study period. These results are similar to trends reported in other studies and included exposure to ACEs, anxiety disorders [27, 50, 52–54], eating/feeding disorders [27], gender dysphoria [55], intentional self-harm [27, 56, 57], mood disorders [27, 50, 52, 57], neurodevelopmental disorders [58], and stand-alone symptoms.

Similar to other reports, we also found a higher risk of MH conditions in males [12, 42, 49, 50, 59, 60] who comprised 52.1% of the study population and 56.5% of those with a MH condition. These higher estimates were due primarily to the neurodevelopmental and disruptive behavior disorders and the corresponding anger/aggression and attentional standalone symptoms. Female comprised 47.9% of the study sample, with 43.4% having a mental health condition with higher prevalence estimates than males in self-harm, depression, eating, somatoform disorders and exposure to ACEs.

In our study, annual prevalence estimates peaked in 2019, prior to the Covid-19 pandemic, similar to the results reported by the Mental Health America screening program [23]. Our data also detected an abrupt decrease in rates for most MH categories for both sexes and race and ethnic groups in the first few months of 2020, coinciding with the beginning of the pandemic quarantine that limited in-person visits, even in EDs from mid- March through mid-April, followed by subsequent increases [61]. The decrease in overall rates in our study persisted for males and white youths, while plateauing for females and the other racial and ethnic groups throughout 2020, with a reversal beginning in 2021 for both sexes and racial and ethnic groups. Exceptions are seen in intentional self-harm and mood disorders with sharp and persistent increases in females while rates in males, plateaued. Similar increases in ED visits for self-harm among adolescent girls was also reported in a meta-analyses of studies that compared ratios of rates of ED visits before and during Covid-19 [62]. This may reflect the global reports of Covid-19 related increases in youth anxiety and depression, especially in girls [63, 64], and suicidality attributed to the pandemic psychosocial stressors [65], Covid viral central nervous system effects [66, 67], and host immunological responses [68].

White youths had higher estimates in all the MH categories except in exposure to ACEs, disruptive behaviors, and psychotic disorders where Hispanic and Black youths had higher rates. Schizophrenia rates for Black youths is shown to be consistently higher than white youths, supporting previous reports of 3-fold higher rates in Black children [69] and adults, [70] although our rate ratios are lower (a peak of 2.38 in the year looking back from May 1, 2021, with a trough of 1.13 for July 1, 2015). In contrast, the rates of suicidality are lower than even epidemiological historical rates and not reflective of the rates of suicide in Black children younger than age 12, a number that is double that of white children [71]. Of note, the higher estimates of exposure to ACEs, seen among Black and Hispanic youths in this study provide some indirect support to the hypotheses linking higher adverse social determinants and risk for psychosis in minority populations. On the other hand, higher prevalence estimates of gender dysphoria among white youths may be indicative of the double stigmatization of gender identity openness in minority populations [72]. The sharp drop in Personality Disorders, in all the groups, may be in part due to the stigma individuals with this diagnoses experience from health professionals as well as the hesitation in recording sensitive information in the EHR [73, 74]. The variation noted at the different sites (Additional File 10, Fig. 2) suggest possible different levels of familiarity by pediatric clinicians in identifying personality disorders. The small number of concept codes and SNOMED CT diagnostic terms, used to extract the data on personality disorders from the EHR, in comparison to other disorders, may also be contributing.

Overall, the variations in prevalence estimates among the different ethnic and racial groups, may in part be due to cultural factors in symptoms expression, stigma, clinician bias, as well as bias in our sample comprising of patients in large medical health systems with access to treatment, and potentially different profiles of neurobiological and psychosocial risk [75, 76]. However, of great interest is that with our expanded typology the smallest variation between the different ethnic and racial groups were noted in the neurodevelopmental, neurocognitive disorders and to a lesser degree among the standalone symptom clusters (where Black youths also had higher rates), suggesting that symptoms may be more objective and less vulnerable to clinician biases that are likely to impact culturally appropriate diagnostic evaluations for minority populations. The standalone symptoms, noted to occur at earlier ages than corresponding cluster diagnostic disorders, further challenge the current diagnostic classifications, such as the DSM, which may be inconsistent with comorbidity and genomic data that indicate shared polygenic risk [77] as well as pleotropic effects reported in mental health, cross-disorder comparisons [78, 79]. Future typology development of the standalone symptoms category could expand on symptom count, ours being parsimonious, including six of the most common ones noted in the pediatric population (see Additional File 7 in Supplement).

The PEDSnet prevalence of 6.8/1000 for delirium, far below rates of 21% [80] to 49% [81] reported in pediatric patients over the course of their hospitalizations, supports reports that it is often not screened for or recognized in the medical setting [82–84]. Catatonia is reported to occur not only in neuropsychiatric disorders but in medical conditions including infections, metabolic disorders, autoimmune disorders, trauma [85–87], however it is often unrecognized and missed [88], reflected in our rate of 0.1/1000 and in the fact that the actual prevalence in medical settings is not known.

The PEDSnet dataset provided information contextualizing patients with mental health visits on service utilization (inpatient, outpatient, ED) at the various sites and diagnostic categories (see Additional Files 9–16, Figs. 1–8). While the rates of mental health patient visits increased approximately 5% in all three service types across the time of the study, the proportion of mental health patients utilizing each service remained approximately the same, with increases in ED captured at the onset of Covid-19. Thus, EHR data may be useful for appropriation of resources that match the use of diverse services on an ongoing basis and capture acute changes, allowing timely shifts of scarce resources.

It is important to note that changes in prevalence estimates of mental health conditions are likely due to multiple factors. For example, prior to the pandemic, the expansion of mental health services within the pediatric medical centers, as well as the increased awareness and implementation of mental health screenings may have played a role in the increased prevalence estimates. The opposite may have occurred at Site D where a decrease in estimated prevalence of mental health conditions in 2014–2015 that coincided with an expansion of pediatric medical beds (increasing the number of all pediatric patients). Likewise, increasing prevalence estimates were seen at each of the 3 services (inpatient, outpatient, ED) up to 2019. The trajectories show steady increases in the outpatient services. The decreases noted in the inpatient services may be due to the increases in pediatric beds (increasing the denominator of all patients with and without MH conditions).

### Limitations

An important limitation of our study is the use of EHR data which comes from PEDSnet institutions, a national network of large academic referral centers, is not random, and is neither nationally representative nor reflective of the general population, as the patients are in medical health care systems with access to treatments. This may bias our sample to more medically complex children or those with more intensive mental health needs.

While the Common data model (OMOP) was applied to standardize data from different sources, our study has the limitations reflecting the current state of EHR data that is not collected for the purpose of research [89]. For example, patients were evaluated in medical clinical settings where standardized instruments were not used to confirm the mental health diagnoses and where there may be under-reporting for mental health disorders [90] not routinely assessed or treated within the medical pediatric home. Additional limitations of EHR data include limited quality and completeness of *ICD* billing codes [11, 12], scarce adaptation of natural language processing and other text processing methods to detect child mental health disorders [91], clinician concern in recording sensitive information that is shared [73] as well capturing social determinants, increasingly recognized as a core component of EHR data [29, 92]. Our study also has limitations stemming from our typology. For example, for social determinants, we did not use any scales and our codes were limited to a single category with 4 clusters (emotional, physical and sexual abuse and neglect). Also, conditions with limited codes (e.g., Personality disorders) may have limited EHR data extraction capabilities.

As the database began in 2009, our estimates of MH visit rates per year showed discontinuities in that first year. To avoid artefacts distorting the results, we decided to drop the 2009 data and focus on the years 2010 and after.

Age when first diagnosis was recorded is likely to be recorded later than age when a diagnosis first emerged. The rising rates of diagnosed disorders could be due to changes in coding behavior among clinicians, which could have occurred with the transition from *ICD-9* to *ICD-10* in 2015. Except for gender dysphoria [55] the monotonic and progressive rates of rise in our study argue against coding practices confounding our findings.

Chart reviews were not done to examine diagnostic codes. The results we report should be considered an assessment of the growing burden of mental health disorder that pediatric health systems must address.

## Conclusions

Our knowledge on the distribution of MH conditions comes largely comes from population surveys [42, 49, 50, 93], disease-specific registries and birth cohorts [59, 94] that are costly to assemble, may not capture the full spectrum of MH conditions in real time or detect trends in changing patient needs.

EHR data also is limited as is its translation to mental health care. In our test-case multi-hospital populations, the new typology we developed for this study allowed the extraction of the full spectrum of MH disorder/symptoms as well as exposure to ACEs, determining rates that approximate those obtained by more costly methods, detected trends over time, and identified groups at risk. More importantly, the clinical informatics methodology used in our study may contribute to breaking down some of the barriers in the translation of aggregated EHR data.

EHR data capturing MH disorders/symptoms and exposure to ACEs, in a systematic way provides a near real-time view of emerging mental health trends which pose a significant challenge to pediatric health systems. These data can rapidly confirm the significance of new symptom patterns and groups at risk at the population level while incorporating early screenings for standalone symptoms by primary care clinicians can lead to early identification and prevention for the individual child at risk. EHR data also have the potential to improve suicide detection where screening questionnaires have short-term predictive validity for future attempts [95–97], identify conditions often unrecognized in the medical setting such as delirium [80] and catatonia [88], and detect chemical and biological terrorist attacks by agents altering mental status [98].

A critical component of our study will be the publicly available EHR-based typology codes that can be used to improve the methods and infrastructure used on collecting and analyzing EHR data for clinical and research purposes as well as for validation purposes. This data can also serve mental health clinicians pursuing training in clinical informatics, and support the call to action to bridge practice and training by Edgcomb and colleagues [29].

The adoption of technologies, such as EHR data, proposed by the National Academy of Medicine [99] could be one of the first steps, that when combined with a health delivery system integrated with the public determinant sectors [100] could provide timely and equitable management of scarce resources. The EHR population health benefits would be further enhanced by designating the growing PEDSnet network as a pediatric mental health surveillance system.

### Electronic supplementary material  (Supplementary material 2. p.28 additional file 11, fig. 3 - could you scroll it to the next page and that will then get all the subsquent ones on their own pages - thank you)

Below is the link to the electronic supplementary material.

Supplementary Material 1

Supplementary Material 2

## Data Availability

Data Sharing Statement and Terminology Code Data Links: The full list of codes and their assignments is publicly available for others to use to further advance research using EHR data (see https://github.com/PEDSnet/MHCC).

## Abbreviations

ACEs: Adverse Childhood Experiences
CI: Confidence Interval
DSM-5: Diagnostic and Statistical Manual of Mental Disorders (fifth edition)
ED: Emergency Department
EHR: Electronic Health Record(s)
ETL: Extract, Transform, Load
IBM: International Business Machine
*ICD-CM*: International Classification of Diseases, Clinical Modification
IQR: Interquartile Range
IRB: Institutional Review Board
MH: Mental Health
MHC: Mental Health Conditions
MHCC: Mental Health Categories Clusters
MHD: Mental Health Disorder(s)
n: Sample size
NSCH: National Survey of Children’s Health
OMOP: Observational Medical Outcome Partnership
PCORI: Patient-Centered Out-comes Research Institute
PCORnet: National Patient Centered Research network
SD: Standard Deviation
SNOMED CT: Systematized Nomenclature of Medicine Clinical Terms
U.S.: United States
