# Supplementary material for: Electronic health records identify timely trends in childhood mental health conditions
Source: Child Adolesc Psychiatry Ment Health. 2023 Sep 14;17:107. doi: 10.1186/s13034-023-00650-7 (PMC10503059; doi:10.1186/s13034-023-00650-7)
Supplement: Supplementary file 1 — Supplementary Material 1 [file 13034_2023_650_MOESM1_ESM.docx]

Supplement 1. STROBE cohort reporting guidelines checklist.

| **Supplement 1: STROBE cohort reporting guidelines checklist** | | | |
| --- | --- | --- | --- |
|  |  | Reporting Item | Page Number |
| **Title and abstract** |  |  |  |
| Title | [#1a](https://www.goodreports.org/reporting-checklists/strobe-cohort/info/#1a) | “Electronic health records identify timely trends in childhood mental health disorders.” | 1 |
| Abstract | [#1b](https://www.goodreports.org/reporting-checklists/strobe-cohort/info/#1b) | Study Design: “Cohort”.  Summary of background, methods, results and conclusions. | 3  3, 4 |
| **Introduction** |  |  |  |
| Background / rationale | [#2](https://www.goodreports.org/reporting-checklists/strobe-cohort/info/#2) | Electronic health records (EHR) have advanced our knowledge of pediatric medical diseases & have the potential to do the same for mental health disorders. | 4, 5, 6 |
| Objectives | [#3](https://www.goodreports.org/reporting-checklists/strobe-cohort/info/#3) | Query of standardized EHR data for full spectrum of mental health disorders, determine ability to detect change over time and compare patients with and without mental health disorders on basis of demographics as well as risk for developing a mental health disorder. | 5, 6, 7 |
| **Methods** |  |  |  |
| Study design | [#4](https://www.goodreports.org/reporting-checklists/strobe-cohort/info/#4) | Cohort study design querying standardized EHR data using a newly developed EHR-based typology. | 7 |
| Setting | [#5](https://www.goodreports.org/reporting-checklists/strobe-cohort/info/#5) | Standardized EHR data from 9 institutions participating in PEDSnet clinical research network from 1/2010 through 11/30/2021. | 7, 8 |
| Eligibility criteria | [#6a](https://www.goodreports.org/reporting-checklists/strobe-cohort/info/#6a) | Patients less than age 21, with 1 or more physician face-to-face visits. | 8 |
| Eligibility criteria | [#6b](https://www.goodreports.org/reporting-checklists/strobe-cohort/info/#6b) | Overall PEDSnet sample with 1^+^physician visit: 7,465,497  Patients younger than age 21: 7,179,578  With a mental health condition: 1,459,230  Without a mental health disorder: 5,720,348 | 8  10, 11  Table 1 |
| Variables | [#7](https://www.goodreports.org/reporting-checklists/strobe-cohort/info/#7) | Mental health disorders (ever diagnosed; annual rates) over 2010-2021, across 9 sites, clinical settings diagnostic categories, patient demographics (age, sex at birth, race/ethnicity, insurance), chronic physical condition. | 6, 7, 8 |
| Data sources / measurement | [#8](https://www.goodreports.org/reporting-checklists/strobe-cohort/info/#8) | Data domains include participating institution, services (inpatient, outpatient, ED), timing of physician visits in calendar year and duration of follow-up, # of chronic physical conditions, mental health diagnoses, patient demographics. | 7, 8  Table 1 |
| Bias | [#9](https://www.goodreports.org/reporting-checklists/strobe-cohort/info/#9) | Proportion rates of mental health disorders were estimated in the full sample using all available data.  Mean follow-up was defined by # of years during which additional visits for any condition occurred even though mean age at entrance into database was similar 6.6-6.5 years. | 9, 10  11 |
| Study size | [#10](https://www.goodreports.org/reporting-checklists/strobe-cohort/info/#10) | EHR data for outpatient, inpatient and ED settings from 2010 through 2021 for patients less than age 21. | 7, 8 |
| Quantitative variables | [#11](https://www.goodreports.org/reporting-checklists/strobe-cohort/info/#11) | Patients with and without a mental health disorder were contrasted on age at first visit, duration of follow-up, chronic physical health conditions and demographics. | 6, 7, 8 |
| Statistical methods | [#12a](https://www.goodreports.org/reporting-checklists/strobe-cohort/info/#12a) | The Standardized difference statistic was used to identify meaningful differences between patients with and without a mental health disorder.  Proportion rates of mental health disorders were estimated in the full sample, using all available data. | 9, 10 |
| Statistical methods | [#12b](https://www.goodreports.org/reporting-checklists/strobe-cohort/info/#12b) | A modified Pediatric Medical comorbidity Algorithm was used to identify patients with chronic physical health conditions.  Median age and interquartile range were computed when patient received their first diagnosis code for that condition and the proportion of diagnosed patients who were male. | 9, 10 |
| Statistical methods | [#12c](https://www.goodreports.org/reporting-checklists/strobe-cohort/info/#12c) | Data for 2009, the first year of the PEDSnet EHR standardized data, was not included due to the number of patients with 1+ physician visits that was much higher than for all the other years. | 10 |
| Statistical methods | [#12d](https://www.goodreports.org/reporting-checklists/strobe-cohort/info/#12d) | Mean follow-up was defined by # of years during which additional visits for any condition occurred even though mean age at entrance into database was similar 6.6-6.5 years. | 11 |
| Statistical methods | [#12e](https://www.goodreports.org/reporting-checklists/strobe-cohort/info/#12e) | Data for 2009, the first year of the PEDSnet EHR standardized data, was not included since the number of patients with 1+ physician visits was much higher than all the other years. | 10 |
| **Results** |  |  |  |
| Participants | [#13a](https://www.goodreports.org/reporting-checklists/strobe-cohort/info/#13a) | Overall PEDSnet sample with 1^+^physician visit: 7,465,497  Patients younger than age 21: 7,179,578  With a mental health condition: 1,459,230  Without a mental health disorder: 5,720,348 | 10, 11  Table 1 |
| Participants | [#13b](https://www.goodreports.org/reporting-checklists/strobe-cohort/info/#13b) | As noted above, inclusion criteria for the overall PEDSnet sample were 1^+^physician visit at any age; Those younger than age 21 were included in this study. | 10, 11 Table 1 |
| Participants | [#13c](https://www.goodreports.org/reporting-checklists/strobe-cohort/info/#13c) | Sample characteristics | Table 1 |
| Descriptive data | [#14a](https://www.goodreports.org/reporting-checklists/strobe-cohort/info/#14a) | Sample characteristics of patients with and without a mental health disorder with regards to age at 1^st^ visit, follow-up duration, sex given at birth, race and ethnicity, year of 1^st^ visit, insurance type, chronic physical conditions. | 10, 11, 12 Table 1 |
| Descriptive data | [#14b](https://www.goodreports.org/reporting-checklists/strobe-cohort/info/#14b) | Race and ethnicity were listed as “other/Unknown “for 11.3% of patients with a mental health disorder and 14.7% for those without.  Insurance type at first visit listed as “other or unknown” for 21.7% of patients with a mental health disorder and 17.7% without a mental health disorder. | Table 1 |
| Descriptive data | [#14c](https://www.goodreports.org/reporting-checklists/strobe-cohort/info/#14c) | Study duration 2010-2021  Mean follow-up duration for patients with a mental health disorder 4.7 years (3.7SD); 2.3 years (3.0 SD) for patients without a mental health disorder. | Table 1  11 |
| Outcome data | [#15](https://www.goodreports.org/reporting-checklists/strobe-cohort/info/#15) | Overall rates of mental health disorders (ever diagnosed; annual rates) with regards diagnostic categories, demographics, sites.  Contrast between patients with mental health diagnoses and those without.  Prevalence of diagnostic categories, clusters with regards to sex and age at first diagnoses. | 10-17  Table 1  18, 19 Table 2  Additional files 9-16. |
| Main results | [#16a](https://www.goodreports.org/reporting-checklists/strobe-cohort/info/#16a) | Risk for a mental health disorder by study year and demographics Rate ratio (95% CI). | 20  Table 3 |
| Main results | [#16b](https://www.goodreports.org/reporting-checklists/strobe-cohort/info/#16b) | Using the Pediatric Medical Complex algorithm, diagnosis terms were assigned body systems, excluding the mental health condition category. | Table 1 |
| Main results | [#16c](https://www.goodreports.org/reporting-checklists/strobe-cohort/info/#16c) | Standardized difference was the statistic used to identify meaningful differences in the 2 distributions (patients with and without mental health disorders). | Table 1 |
| Other analyses | [#17](https://www.goodreports.org/reporting-checklists/strobe-cohort/info/#17) | To identify risk factors for any type of mental health disorder, we fit a Poisson regression. | 10 |
| **Discussion** |  |  |  |
| Key results | [#18](https://www.goodreports.org/reporting-checklists/strobe-cohort/info/#18) | Key results are summarized. | 10-21 |
| Limitations | [#19](https://www.goodreports.org/reporting-checklists/strobe-cohort/info/#19) | EHR data from PEDSnet institutions, a national network of large academic referral centers is not random, nor representative of the general population, as the patients are in a medical setting with access to evaluations and treatments. | 26, 27 |
| Interpretation | [#20](https://www.goodreports.org/reporting-checklists/strobe-cohort/info/#20) | Our results indicate that mental health diagnoses derived from standardized EHR records can capture the full spectrum of mental health disorders and these rates approximate those obtained by more costly methods. Our results also supported the growing burden of mental health disorders (identified by other groups) over the 11-year time span, identified groups at risk, and detected trends during the Covid-19 pandemic. | 21, 22, 23, 24, 25. |
| Generalizability | [#21](https://www.goodreports.org/reporting-checklists/strobe-cohort/info/#21) | Similarities and differences of results with other studies is discussed. Limitations to the generalizability is noted since our sample is from academic centers and not reflective of the general population. | 21, 22, 23, 24, 25 |
| **Other Information** |  |  |  |
| Funding | [#22](https://www.goodreports.org/reporting-checklists/strobe-cohort/info/#22) | Award number RI-CRN-2020-007 from the Patient Centered Out-comes research Institute (PCORI). | 31 |

von Elm E, Altman DG, Egger M, Pocock SJ, Gotzsche PC, Vandenbroucke JP. The Strengthening the Reporting of Observational Studies in Epidemiology (STROBE) Statement: guidelines for reporting observational studies.

The STROBE checklist is distributed under the terms of the Creative Commons Attribution License CC-BY. This checklist can be completed online using <https://www.goodreports.org/>, a tool made by the [EQUATOR Network](https://www.equator-network.org) in collaboration with [Penelope.ai](https://www.penelope.ai)
