# Supplementary material for: Electronic health records identify timely trends in childhood mental health conditions
Source: Child Adolesc Psychiatry Ment Health. 2023 Sep 14;17:107. doi: 10.1186/s13034-023-00650-7 (PMC10503059; doi:10.1186/s13034-023-00650-7)
Supplement: Supplementary file 2 — Supplementary Material 2 [file 13034_2023_650_MOESM2_ESM.docx]

Additional File 2: EHR-based Pediatric Mental Health Conditions Typology

Additional File 2: Electronic Health Record-based pediatric mental health typology: categories, clusters, and condition examples

Category	Cluster	Condition Examples
**Adverse Childhood Experiences**	**Emotional Abuse**	Emotional abuse of child
		Emotional deprivation of child
		Child victim of emotional or psychological abuse
	**Neglect**	Child neglect
		Child abandonment
		Nutritional maltreatment of child
	**Physical Abuse**	Battered baby
		Child victim of physical abuse
		Munchausen's by proxy
	**Sexual Abuse**	Child sex abuse
		Victim of human trafficking
		Victim of child sexual exploitation
**Anxiety Disorders**	***Anxiety Disorder**	Generalized anxiety disorder
		Separation anxiety disorder
		Social anxiety disorder
	***Obsessive-Compulsive Problems**	Obsessive compulsive disorder
		Body dysmorphic disorder

Additional File 2: Electronic Health Record-based pediatric mental health typology: categories, clusters, and condition examples		
**Category**	**Cluster**	**Condition Examples**
		Trichotillomania
	*Somatoform Disorders	Somatic symptom disorder
		Conversion disorder
		Factitious disorder
	*Stress Disorders	Acute stress disorder
		Post-traumatic stress disorder
		Reactive attachment disorder
*Disruptive Behavioral Disorders	Conduct Disorder	Conduct disorder, childhood-onset
		Conduct disorder, adolescent-onset
		Conduct disorder, undifferentiated type
	Impulse Control Disorder	Pyromania
		Kleptomania
		Intermittent explosive disorder
	Oppositional-Defiant Disorder (ODD)	ODD concurrent with chronic irritability/anger
		ODD with chronic irritability/anger with limited prosocial emotions
		ODD without chronic irritability/anger with limited prosocial emotions
*Eating and Feeding	Avoidant/Restrictive Food Intake	Anorexia nervosa
		Bulimia nervosa

Additional File 2: Electronic Health Record-based pediatric mental health typology: categories, clusters, and condition examples		
**Category**	**Cluster**	**Condition Examples**
**Problems**		Binge eating disorder
	**Other Feeding/Eating Problems**	Pica
		Rumination disorder
		Psychogenic vomiting
***Elimination Disorders**	**Encopresis**	Encopresis with constipation and overflow incontinence
		Soiling encopresis
		Functional encopresis
	**Enuresis**	Nocturnal enuresis
		Daytime enuresis
		Intermittent urinary incontinence
**Gender Dysphoria/Sexual Dysfunction**	***Gender Dysphoria**	Gender dysphoria in children
		Gender dysphoria in adolescents and adults
		Unspecified gender dysphoria
	***Paraphilia**	Voyeuristic disorder
		Exhibitionistic disorder
		Pedophilic disorder
	***Sexual Dysfunction**	Delayed ejaculation
		Erectile disorder
		Substance/medication induced

Additional File 2: Electronic Health Record-based pediatric mental health typology: categories, clusters, and condition examples

Category	Cluster	Condition Examples
**Intentional Self-Harm**	**Parasuicidality**	Deliberate self-cutting
		Self-injury by cigarette burn
		Biting self
	**Suicidality**	Suicidal ideation
		Suicide attempt
		Other preparatory acts to kill self
**Mood Disorders**	***Bipolar Disorders**	Bipolar I disorder
		Bipolar II disorder
		Cyclothymia
	***Major Depression**	Major depression, single episode
		Major depression, recurrent
		Substance/ medication induced
	**Minor Depression**	Persistent depressive disorder (dysthymia)
		Reactive depression
		Adjustment disorder with depressed mood
**Neurocognitive Disorders**	**Catatonia**	Catatonia due to medical condition
		Catatonia due to psychiatric disorder
		Substance/ medication-induced/ withdrawal
		Delirium due to multiple etiologies

Additional File 2: Electronic Health Record-based pediatric mental health typology: categories, clusters, and condition examples

Category	Cluster	Condition Examples
	***Delirium**	Delirium due to another medical condition
		Substance/ medication-induced/ withdrawal delirium
	**Encephalopathy**	N-methyl d-aspartate receptor antibody
		Neuropsychiatric lupus erythematosus
		Encephalitis
***Neurodevelopmental Disorders**	**Academic Developmental Disorder**	Developmental reading disorder
		Developmental arithmetic disorder
		Developmental expressive writing disorder
	**Attention-Deficit, Hyperactivity Disorder (ADHD)**	ADHD, inattentive subtype
		ADHD, hyperactive-impulsive subtype
		ADHD, combined subtypes
	**Autism Spectrum Disorder**	Autism disorder
		Asperger’s disorder
		Neurogenetic disorders with autistic features
	**Communication/Motor Disorders**	Language disorder (receptive, expressive)
		Speech sound disorder
		Childhood-onset fluency disorder (stuttering)
	**Intellectual Disabilities**	Mild, moderate, severe intellectual disability
		Global developmental delay

Additional File 2: Electronic Health Record-based pediatric mental health typology: categories, clusters, and condition examples		
**Category**	**Cluster**	**Condition Examples**
		Neurogenetic disorders with intellectual disability
***Personality Disorders**	**Personality Disorders**	Paranoid personality disorder
		Borderline personality disorder
		Avoidant personality disorder
***Psychotic Disorders**	**Psychotic Disorder**	Brief psychotic disorder
		Post-partum psychoses
		Substance/medication induced
	**Schizoaffective Disorder**	Schizoaffective disorder, bipolar type
		Schizoaffective disorder, depressive type
		Affective psychosis
	**Schizophrenia**	Paranoid schizophrenia
		Undifferentiated schizophrenia
		Catatonic schizophrenia
***Sleep-Wake Disorders**	**Hypersomnia**	Hypersomnia
		Narcolepsy
		Recurrent hypersomnia
	**Insomnia**	Insomnia
		Chronic insomnia
		Not getting enough sleep

Additional File 2: Electronic Health Record-based pediatric mental health typology: categories, clusters, and condition examples		
**Category**	**Cluster**	**Condition Examples**
	**Parasomnias**	Non-rapid eye movement sleep Arousal disorder
		Nightmare disorders
		Restless legs syndrome
**Standalone Symptoms**	**Anger/Aggression**	Physical aggression
		Irritability and anger
		Bullying
	**Anxiety Symptoms**	Feeling nervous
		Worried
		Fear
	**Attention Symptoms**	Distractibility
		Inattention
		Poor concentration
	**Depressive Symptoms**	Feeling unhappy
		Anhedonia
		Loss of interest
	**Hallucinations**	Hallucinations
		Auditory hallucinations
		Visual hallucinations
	**Alcohol**	Alcohol use disorder

**Additional File 2: Electronic Health Record-based pediatric mental health typology: categories, clusters, and condition examples**		
**Category**	**Cluster**	**Condition Examples**
***Substance Use/ Dependence**		Alcohol dependence
		Alcohol intoxication/withdrawal
	**Opioid-Related Disorders**	Opioid use disorder
		Opioid dependence
		Opioid intoxication/withdrawal
	**Other Substances (e.g., hallucinogen, sedative/hypnotic)**	Other substances use disorder
		Other substances dependence
		Polysubstance use/dependence
	**Tetrahydrocannabinol**	THC use disorder
		THC dependence
		THC intoxication/withdrawal
	**Tobacco**	Tobacco use disorder
		Tobacco dependence
		Tobacco intoxication/withdrawal
**Tic Disorders**	**Tic Disorders**	Acute onset tic disorder
		Chronic tic disorder
		Tourette disorder
*Corresponding DSM-5 diagnostic groups		

Additional File 2: Electronic Health Record-based pediatric mental health typology: categories, clusters, and condition examples		
**Category**	**Cluster**	**Condition Examples**

ADDITIONAL FILES 3-8: DEVELOPMENT OF EHR-BASED PEDIATRIC MENTAL HEALTH TYPOLOGY-METHODS

Electronic health records (EHRs) present an opportunity to collect epidemiologic and treatment information on patient populations at scale and efficiently (i.e., quickly and with low cost), enhancing clinical research in this area.[1,2] As reviewed by Williams and colleagues[3], the raw EHR data containing structured data (clinical codes for problems) and unstructured data (free text) require the construction of "code sets". This process involves assembling a set of clinical codes that represents a single concept such as a diagnosis. Once constructed, these code sets can be used to query and extract data from the EHR database.

The development of the EHR-based pediatric mental health typology, used in this study, started by assembling a set of clinical codes from the Systematized Nomenclature of Medicine Clinical Terms (SNOMED CT) [4,5]. SNOMED CT, is a systematically organized computer processable collection of medical terms providing codes, synonyms and definitions used in clinical documentation, was used to provide the core general terminology for the electronic health records. It allows a consistent way to index, store and retrieve medical data across specialties and sites of care [4,5]. SNOMED CT has four primary core components:

1. Concept Codes- numerical codes that identify clinical terms, primitive or defined, organized in hierarchies
2. Descriptions – textual descriptions of Concept Codes
3. Relationships – relationships between Concept Codes that have a related meaning
4. Reference Set – used to group Concepts or Descriptions into sets, including reference sets and cross-maps to other classifications and standards

SNOMED CT can cross-map to other international standards and classifications such as the International Statistical Classification of Diseases and Related Problems (*ICD*) code set, maintained by the World Health Organization. [6] This is the most widely used statistical classification system for diseases in the world that is used as a diagnostic tool for epidemiology and health management.[6] More specifically, we used SNOMED CT to cross-map to The International Classification of Diseases, Clinical Modification (*ICD-CM*), an adaption created by the U.S. National Center for Health Statistics, updated annually, and used in assigning diagnostic and procedure codes associated with inpatient, outpatient and physician utilization in the U.S. [7] Both SNOMED CT and *ICD* use standardized definitions and form a common medical language used within the electronic health record (EHR) systems. [8] SNOMED CT enables information input into an EHR system during the course of patient care, while ICD facilitates information retrieval, or output, for secondary data purposes.[8]

Some important considerations for our study included the *ICD* code revisions. In 2015 US healthcare transition to the current International Classification of Diseases, Tenth Revision, Clinical Modifications (*ICD-10-CM*) coding,[9] This reclassified child mental health disorders were under new psychiatric diagnostic groups and diagnosis codes for individual disorders, that differed from the previous classifications (*ICD-9-CM*). [10] Another consideration was the differences in granularity, emphasis and organizing principles between SNOMED CT and *ICD-10-CM*, where is not always possible to have a one-to-one map between a SNOMED CT concept and an *ICD-10-CM* code.

A team of six clinicians (four child and adolescent psychiatrists (JE, AP, KP, RP) and two general pediatricians (CF, LU) worked closely at each step of the development. This started with the identification of categories and clusters of the major pediatric mental health disorders and development of code sets for each of the categories, clusters, and symptoms. These code sets were checked for omissions and commissions and alignment with the Diagnostic and Statistical Manual of Mental Disorders (fifth edition) DSM-5 [11] and revised accordingly.

Condition categories and clusters code development

A total of 4047 SNOMED-CT codes were identified for the 16 mental health condition categories and 49 clusters that were mapped on to 567 *ICD 10* codes. These were used in the epidemiology analyses of the clusters overall and over time.

The tables below include the number of SNOMED-CT and *ICD-10* codes for each of the 16 categories (**Additional File 3**) and the 49 clusters (**Additional File 4**).

ADDITIONAL FILE 3. Mental Health Condition Categories and corresponding number of SNOMED-CT and *ICD-10* codes

	**SNOMED-CT**	***ICD-10***
Adverse Childhood Experiences	107	11
Anxiety Disorders	343	87
Disruptive Behavior Disorders	54	18
Eating and Feeding Disorders	40	11
Elimination Disorders	24	3
Gender Dysphoria/Sexual Dysfunction	111	29
Intentional Self-Harm/Suicidality	1251	54
Mood Disorders	307	37
Neurocognitive Disorders	288	74
Neurodevelopmental Disorders	559	61
Personality Disorders	49	24
Psychotic Disorders	220	54
Sleep-Wake Disorders	102	6
Standalone Symptoms	393	15
Substance Use and Dependence	167	76
Tic Disorders	32	7

**Additional File 4. Mental Health Clusters and corresponding number of SNOMED-CT and *ICD-10* codes**			
**CATEGORY**	**CLUSTER**	**SNOMED-CT**	***ICD-10***
Adverse Childhood Experiences	Emotional Abuse	17	2
Adverse Childhood Experiences	Neglect	22	7
Adverse Childhood Experiences	Physical Abuse	41	1
Adverse Childhood Experiences	Sexual Abuse	27	1
Anxiety Disorders	Anxiety Disorder	120	31
Anxiety Disorders	OCD	20	6
Anxiety Disorders	Somatoform Disorder	129	29
Anxiety Disorders	Stress Disorder	74	21
Disruptive Behavior Disorders	Conduct Disorder	29	10
Disruptive Behavior Disorders	Impulse Control Disorder	19	7
Disruptive Behavior Disorders	Oppositional Defiant Disorder	6	1
Eating and Feeding Disorders	Avoidant/Restrictive Food Intake	27	9
Eating and Feeding Disorders	Other Eating and Feeding Disorder	13	2
Elimination Disorders	Encopresis	14	2
Elimination Disorders	Enuresis	10	1
Gender Dysphoria/Sexual Dysfunction	Gender Dysphoria	25	7
Gender Dysphoria/Sexual Dysfunction	Paraphilia	53	10
Gender Dysphoria/Sexual Dysfunction	Sexual Dysfunction	33	12
Intentional Self-Harm/Suicidality	Parasuicidality	66	9
Intentional Self-Harm/Suicidality	Suicidality	1185	45
Mood Disorders	Bipolar Disorder	121	7
Mood Disorders	Major Depression	130	10
Mood Disorders	Minor Depression	56	20

Neurocognitive Disorders	Catatonia	3	2
Neurocognitive Disorders	Delirium	84	41
Neurocognitive Disorders	Encephalopathy	201	31
Neurodevelopmental Disorders	Academic Developmental Disorder	22	7
Neurodevelopmental Disorders	ADHD	16	5
Neurodevelopmental Disorders	Autism Spectrum Disorder	27	7
Neurodevelopmental Disorders	Communication/Motor Disorder	72	9
Neurodevelopmental Disorders	Intellectual Disability	422	33
Personality Disorders	Personality Disorder	49	24
Psychotic Disorders	Psychotic Disorder	141	34
Psychotic Disorders	Schizoaffective Disorder	8	6
Psychotic Disorders	Schizophrenia	71	14
Sleep-Wake Disorders	Hypersomnia	29	2
Sleep-Wake Disorders	Insomnia	35	1
Sleep-Wake Disorders	Parasomnias	38	3
Standalone Symptoms	Anger/Aggression	89	6
Standalone Symptoms	Anxiety Symptoms	189	3
Standalone Symptoms	Attention Symptoms	39	1
Standalone Symptoms	Depressive Symptoms	19	1
Standalone Symptoms	Hallucinations	57	4
Substance Use and Dependence	Alcohol	28	8
Substance Use and Dependence	Opioid Related	14	4
Substance Use and Dependence	Other Substances	99	46
Substance Use and Dependence	THC	17	6
Substance Use and Dependence	Tobacco	9	12
Tic Disorders	Tic Disorder	32	7

In Additional File **5** and Additional File **6,** we highlight the Adverse Childhood Experience Category, Emotional Abuse Cluster as an example. 17 SNOMED-CT concept codes were identified that mapped to 2 *ICD 10* codes.

Additional File 5. Adverse childhood experience category- Emotional abuse cluster SNOMED-CT code set

concept_id	concept_code	concept_name	vocabulary_id	cluster	Category
**43020442**	207871000119100	Adult victim of emotional abuse	SNOMED	Emotional Abuse	Adverse Childhood Experiences
**43021802**	473453008	Child victim of psychological or emotional abuse	SNOMED	Emotional Abuse	Adverse Childhood Experiences
**4161447**	371773006	Emotional abuse	SNOMED	Emotional Abuse	Adverse Childhood Experiences
**436001**	371774000	Emotional abuse of adult	SNOMED	Emotional Abuse	Adverse Childhood Experiences
**439395**	371775004	Emotional abuse of child	SNOMED	Emotional Abuse	Adverse Childhood Experiences
**4072656**	242047002	Emotional abuse of disabled person	SNOMED	Emotional Abuse	Adverse Childhood Experiences
**4149657**	278707004	Emotional deprivation of child	SNOMED	Emotional Abuse	Adverse Childhood Experiences
**4192665**	39465007	Emotional deprivation syndrome	SNOMED	Emotional Abuse	Adverse Childhood Experiences
**4193573**	313217007	History of emotional abuse	SNOMED	Emotional Abuse	Adverse Childhood Experiences
**45757170**	10760941000119102	Psychological abuse complicating childbirth	SNOMED	Emotional Abuse	Adverse Childhood Experiences
**45757171**	10760981000119107	Psychological abuse complicating pregnancy	SNOMED	Emotional Abuse	Adverse Childhood Experiences
**4319168**	95925006	Psychologically abused woman	SNOMED	Emotional Abuse	Adverse Childhood Experiences

**37108776**	12399131000119105	Suspected victim of child emotional abuse	SNOMED	Emotional Abuse	Adverse Childhood Experiences
**44783767**	697951004	Suspected victim of emotional abuse	SNOMED	Emotional Abuse	Adverse Childhood Experiences
**4310558**	423402005	Victim of consistent negative messages	SNOMED	Emotional Abuse	Adverse Childhood Experiences
**443549**	419916003	Victim of emotional abuse	SNOMED	Emotional Abuse	Adverse Childhood Experiences
**4026164**	225825002	Victim of verbal abuse	SNOMED	Emotional Abuse	Adverse Childhood Experiences

Additional File 6. Adverse childhood experience clusters SNOMED-CT code sets and corresponding *ICD 10* codes

concept_id	concept_code	concept_name	vocabulary_id	cluster	category	icd10_code	icd10_name
**4161447**	371773006	Emotional abuse	SNOMED	Emotional Abuse	Adverse Childhood Experiences	T74.3	Psychological abuse
**439395**	371775004	Emotional abuse of child	SNOMED	Emotional Abuse	Adverse Childhood Experiences	Z62.3	Hostility towards and scapegoating of child
**4160623**	371776003	Neglect or abandonment	SNOMED	Neglect	Adverse Childhood Experiences	T74.0	Neglect or abandonment
**4160623**	371776003	Neglect or abandonment	SNOMED	Neglect	Adverse Childhood Experiences	Y06	Neglect and abandonment
**4160623**	371776003	Neglect or abandonment	SNOMED	Neglect	Adverse Childhood Experiences	Y06.0	Neglect and abandonment by spouse or partner
**4160623**	371776003	Neglect or abandonment	SNOMED	Neglect	Adverse Childhood Experiences	Y06.1	Neglect and abandonment by parent
**4160623**	371776003	Neglect or abandonment	SNOMED	Neglect	Adverse Childhood Experiences	Y06.2	Neglect and abandonment by acquaintance or friend
**4160623**	371776003	Neglect or abandonment	SNOMED	Neglect	Adverse Childhood Experiences	Y06.8	Neglect and abandonment by other specified persons
**4160623**	371776003	Neglect or abandonment	SNOMED	Neglect	Adverse Childhood Experiences	Y06.9	Neglect and abandonment by unspecified person
**4302066**	418506006	Physical abuse	SNOMED	Physical Abuse	Adverse Childhood Experiences	T74.1	Physical abuse

**443080**	213017001	Sexual abuse	SNOMED	Sexual Abuse	Adverse Childhood Experiences	T74.2	Sexual abuse
**381537**	17496003	Organic anxiety disorder	SNOMED	Anxiety Disorder	Anxiety Disorders	F06.4	Organic anxiety disorder

Standalone symptom code development.

Initially names for a parsimonious number of symptoms common in pediatric mental health disorders were identified. Initially this included 7 symptoms: anger, anxiety, attention, depressive, hallucinations, delusions, stress. The symptom cluster of delusions was deleted to focus on the most common symptoms in pediatrics, leaving 6.

596 SNOMED Ancestor concept names were found to correspond to these 6 symptoms. After reviews and discussions, those concept names that corresponded to mental health disorders captured in other clusters were deleted. The final number of SNOMED concept names was 392 and these mapped to 26 *ICD 10* codes below.

Additional File 7. Mental Health Symptoms corresponding *ICD-10* codes

Cluster	ICD-10 Code	Description
Attention symptoms	R41840	Attention and concentration deficit
Hallucinations	R440	Auditory hallucinations
Hallucinations	R441	Visual hallucinations
Hallucinations	R442	Other hallucinations
Hallucinations	R443	Hallucinations, unspecified
Anxiety symptoms	R450	Nervousness and agitation
Anxiety symptoms	R451	Restlessness and agitation
Depressive symptoms	R452	Unhappiness
Depressive symptoms	R453	Demoralization and apathy
Anger	R454	Irritability and anger
Anger	R455	Hostility
Anger	R456	Violent behavior
Stress	R457	State of emotional shock and stress, unspecified
Depressive symptoms	R4581	Low self esteem
Anxiety symptoms	R4582	Worries
Depressive symptoms	R4583	Excessive crying of child, adolescent, or adult
Depressive symptoms	R4584	Anhedonia

Depressive symptoms	R4585	Homicidal and suicidal ideations
Depressive symptoms	R45850	Homicidal ideations
Depressive symptoms	R45851	Suicidal ideations
Depressive symptoms	R4586	Emotional lability
Attention symptoms	R4587	Impulsiveness
Depressive symptoms	R4588	Nonsuicidal self-harm
Attention symptoms	R463	Overactivity
Stress	Z733	Stress, not elsewhere classified
Depressive symptoms	T1491	Suicide attempt

ALIGNMENT OF PEDSnet TYPOLOGY AND DMS-5 DIAGNOSTIC GROUPS

The PEDSnet Typology is composed of 16 pediatric mental health condition categories and 49 clusters. The Diagnostic and Statistical Manual of Mental Disorders, Fifth Edition (DSM-5), [11] an analogue to the *ICD*, that is limited to psychiatric disorders and used primarily in the U.S, is composed of 19 diagnostic groups. **In Additional File 8,** the alignment of PEDSnet Typology condition categories and clusters and DSM-5 diagnostic groups is summarized.

**Additional File 8. Alignment of PEDSnet Typology and DSM-5 diagnostic groups**				
**DSM-5**			**PEDSnet TYPOLOGY**	
**Diagnostic Categories (# 1-19) & Other Conditions**			**Categories**	**Clusters**
	Other conditions that may be a focus of Clinical attention		Adverse Childhood Experiences	Emotional Abuse
				Neglect
				Physical Abuse
				Sexual Abuse
5.	Anxiety Disorders		Anxiety Disorders	Anxiety Disorder
6.	Obsessive-Compulsive & related disorders			Obsessive-Compulsive Problems
9.	Somatic Symptoms & related disorders			Somatoform Disorders
8.	Dissociative Disorders			
7.	Trauma & stress related disorders			Stress Disorders
15.	Disruptive, Impulse-Control and Conduct Disorder		Disruptive Behavioral Disorders	Conduct Disorder
				Impulse Control Disorder
				Oppositional-defiant disorder
10.	Feeding & eating disorders	Anorexia Nervosa	Eating & feeding problems	Avoidant/Restrictive Food Intake
		Bulimia Nervosa		
		Avoidant/Restrictive Food Intake Disorder		
		Binge Eating Disorder		
		Pica		Other feeding/eating problems
		Rumination Disorder		
11.	Elimination disorders		Elimination Disorders	Encopresis

				Enuresis
14.	Gender dysphoria		Gender dysphoria / Sexual dysfunction	Gender Dysphoria
19.	Paraphilic disorder			Paraphilia
13.	Sexual Dysfunction			Sexual Dysfunction
			Intentional Self-Harm	Parasuicidality
				Suicidality
3.	Bipolar & Related Disorders		Mood Disorders	Bipolar Disorders
4.	Depressive Disorders			Major Depression
		Persistent Depressive Disorder (Dysthymia)		Minor Depression
		Premenstrual Dysphoric Disorder		
		Disruptive Mood Dysregulation		
17.	Neurocognitive Disorders		Neurocognitive Disorders	Catatonia
		Delirium		Delirium
				Encephalopathy
		Dementias		
		Infections		
		Prion Diseases		
1.	Neurodevelopmental Disorders	Specific learning disorder	Neurodevelopmental Disorders	Academic developmental disorder
				ADHD
				Autism spectrum disorder
		Communication disorders		Communication/ Motor Dis
		Motor Disorders		
		Intellectual Disabilities		Intellectual disability
18.	Personality Disorders		Personality Disorders	Personality Dis
		Paranoid		Paranoid
		Schizoid		
		Schizotypal		
		Antisocial		
		Borderline		Borderline
		Histrionic		
		Narcissistic		
		Avoidant		Avoidant
		Dependent		
		Obsessive-Compulsive		

2.	Schizophrenia Spectrum & Other Psychotic Disorders		Psychotic Disorders	Psychotic Disorder
				Schizoaffective Disorder
				Schizophrenia
		Delusional disorder		
		Schizophreniform disorder		
		Schizotypal personality disorder		
12.	Sleep-Wake Disorders		Sleep-Wake Disorders	Hypersomnia
				Insomnia
				Parasomnias
		Narcolepsy		
		Breathing-related disorders		
			Standalone Symptoms	Anger/Aggression
				Anxiety
				Attention
				Depressive
				Hallucinations
16.	Substance-Related & Addictive Disorders	Alcohol-related disorders	Substance Use / Dependence	Alcohol
		Cannabis-Related Disorders		Tetrahydrocannabinol (THC)
		Phencyclidine		
		Other hallucinogen use		Other substances (e.g., hallucinogens, sedative/hypnotics)
		Sedative, hypnotic, or anxiolytic		
		Opioid-Related Disorders		Opioid-related disorders
		Tobacco		Tobacco
		Caffeine-related disorders		
		Non-Substance related (gambling)		
			Tic Disorders	Tic Disorders

Abbreviations: Abbreviations: ADHD, attention-deficit/hyperactivity disorder; OCD, obsessive-compulsive disorder; ODD, oppositional defiant disorder; THC, tetrahydrocannabinol

Light Blue: DSM conditions with different terminology for PEDS net clusters

Dark Blue: DMS diagnostic categories not included in PEDSnet categories or clusters. The ones relevant to pediatric age ranges could be considered in future revisions.

a. Dementias – more relevant in adults

b. Infections – relevant in all ages and relevant for psychiatric disorders.
c. Prion disease – relevant in all ages and relevant for psychiatric disorders.
d. Breathing-related sleep disorders- relevant in all ages and relevant for psychiatric disorders.
e. Non-substance related addictive disorders (e.g., gambling). Relevant addictive disorders such as videos should be considered in future revisions of PEDSnet typology.

Dark Green: Categories and clusters that we added in the PEDSnet typology that are not in the DSM-5 diagnostic categories.

Adverse Childhood Experiences. These clusters are included in the DSM-5 section (not defined as a diagnostic category) called "Other conditions that may be a focus of clinical attention". These have been shown to have a significant impact on health outcomes. [12]

Catatonia - In DSM-5, catatonia is not an independent diagnostic class but associated with psychiatric and medical conditions including infections, metabolic disorders, autoimmune disorders, and trauma. [13-15] Catatonia is often not recognized in medical conditions and needs to be emphasized given the available treatments and significant morbidity and mortality when untreated. [16]

Encephalopathy – In autoimmune encephalopathies, 90% of patients have psychiatric symptoms difficult to distinguish from a primary psychiatric disorder. [17] As more assays are developed for antibodies for CNS proteins, this cluster will likely become more prevalent. Also, treatments, available for some of these such NMDA-R ab can be curative.

Standalone symptoms are included because they are not specific to any single disorder and may be early manifestations of some disorders.

Tic disorders – In DSM-5 are under motor disorders. We developed a new category since tics may be related to other conditions (e.g., OCD)

REFERENCES

1. Cowie MR, Blomster JI, Curtis LH, et al. Electronic health records to facilitate clinical research. *Clin Res Cardiol*. Jan 2017;106(1):1-9. doi:10.1007/s00392-016-1025-6
2. Wasserman RC. Electronic medical records (EMRs), epidemiology, and epistemology: reflections on EMRs and future pediatric clinical research. *Acad Pediatr*. 2011 Jul-Aug 2011;11(4):280-7. doi:10.1016/j.acap.2011.02.007
3. Williams R, Kontopantelis E, Buchan I, Peek N. Clinical code set engineering for reusing EHR data for research: A review. *J Biomed Inform*. 06 2017;70:1-13. doi:10.1016/j.jbi.2017.04.010
4. Kostick K. SNOMED CT integral part of quality EHR documentation. *J AHIMA*. Oct 2012;83(10):72-5.
5. Organzation IHTSD. Use SNOMED CT. www.snomed.org
6. Organization WH. International Classification of Diseases. https://www.who.int/home/cms-decommissioning}p.MsoNormal, li.MsoNormal, div.MsoNormalfont-size:11.0ptfont-size:11.0ptmso-ansi-font-size:11.0ptmso-bidi-font-size:11.0pt}div.WordSection1
7. CDC. ICD-10-CM. https://www.cdc.gov/nchs/icd/icd10cm.htm
8. Bowman S. Coordinating SNOMED-CT and ICD-10. *J AHIMA*. 2005 Jul-Aug 2005;76(7):60-1.
9. Reeves SL, Freed GL. Problems With Quality Measurement Using International Statistical Classification of Diseases, Tenth Revision, Clinical Modification: The Elephant No One Knows Is in the Room. *JAMA Pediatr*. 06 2019;173(6):515-516. doi:10.1001/jamapediatrics.2019.0844
10. CDC. ICD-CM-9.
11. Association AP. *Diagnostic and Statistical Manual of Mental Disorders, Fifth Edition*. 5th ed. American Psychiatric Association; 2013.
12. Jones CM, Merrick MT, Houry DE. Identifying and Preventing Adverse Childhood Experiences: Implications for Clinical Practice. *JAMA*. Jan 07 2020;323(1):25-26. doi:10.1001/jama.2019.18499
13. Denysenko L, Sica N, Penders TM, et al. Catatonia in the medically ill: Etiology, diagnosis, and treatment. The Academy of Consultation-Liaison Psychiatry Evidence-Based Medicine Subcommittee Monograph. *Ann Clin Psychiatry*. May 2018;30(2):140-155.
14. Carroll BT, Anfinson TJ, Kennedy JC, Yendrek R, Boutros M, Bilon A. Catatonic disorder due to general medical conditions. *J Neuropsychiatry Clin Neurosci*. 1994;6(2):122-33. doi:10.1176/jnp.6.2.122
15. Smith JH, Smith VD, Philbrick KL, Kumar N. Catatonic disorder due to a general medical or psychiatric condition. *J Neuropsychiatry Clin Neurosci*. 2012;24(2):198-207. doi:10.1176/appi.neuropsych.11060120
16. Dhossche DM, Wachtel LE. Catatonia is hidden in plain sight among different pediatric disorders: a review article. *Pediatr Neurol*. Nov 2010;43(5):307-15. doi:10.1016/j.pediatrneurol.2010.07.001
17. Kayser MS, Titulaer MJ, Gresa-Arribas N, Dalmau J. Frequency and characteristics of isolated psychiatric episodes in anti–N-methyl-d-aspartate receptor encephalitis. *JAMA Neurol*. Sep 01 2013;70(9):1133-9. doi:10.1001/jamaneurol.2013.3216

DATA BY SITE, SERVICE & MH CONDITION CATEGORIES, USING EHR-BASED PEDIATRIC MENTAL HEALTH TYPOLOGY

Additional File 9, Figure 1. *Annual rolling percentages of patients <21 years-old with an identified mental health condition/all patients, by site, 2010 to 2023.*
Annual rolling proportions were computed each month using denominators that were the count of patients in each of the 9 sites (with a visit in any inpatient, outpatient, ED setting) in the prior 12 months. The numerator was the count of distinct patients with at least one mental health condition during this same time-period.

Additional File 10, Figure 2. *Annual rolling percentages of patients <21 years-old with an identified mental health condition/all patients, by condition category and site, 2010 to 2023.*
Annual rolling proportions were computed each month using denominators that were the count of patients in each of the 9 sites (with a visit in any inpatient, outpatient, ED setting) in the prior 12 months, in each of the 16 diagnostic categories. The numerator was the count of distinct patients with at least one visit for a mental health condition of that type during this same time-period.

Additional File 11, Figure 3. *Annual rolling percentages of patients <21 years-old with an identified mental health condition/all patients, by Service (Inpatient, outpatient, ED), 2010 to 2023.*
Annual rolling proportions were computed each month using denominator that were the count of all patients (not necessarily MH) in each of the 3 services (1+ visit in inpatient, outpatient, ED) in the prior 12 months. The numerator was the count of distinct patients with at least one mental health condition during this same time-period.

Additional File 12, Figure 4. *Annual rolling percentages of patients <21 years-old with an identified mental health condition/all patients, by Service (Inpatient, outpatient, ED), and site, 2010 to 2023.*
Annual rolling proportions were computed each month using denominator that were the count of all patients (not necessarily MH) in each of the 3 services (1+ visit in inpatient, outpatient, ED) in the prior 12 months. The numerator was the count of distinct patients with at least one visit for a mental health condition of that type during this same time-period.

Additional File 13, Figure 5. *Annual rolling percentages of patients <21 years-old with an identified mental health disorder by Service (Inpatient, outpatient, ED), 2010 to 2023.*
Annual rolling proportions were computed each month using denominator that were the count of all patients with 1+ mental health condition visit, in any of the 3 services (inpatient, outpatient, ED) in the prior 12 months. The numerator was the count of distinct patients with MH condition in each of the 3 services.

Additional File 14, Figure 6. *Annual rolling percentages of patients <21 years-old with an identified mental health condition by Service (Inpatient, outpatient, ED), and site, 2010 to 2023.*
Annual rolling proportions were computed each month using denominator that were the count of all patients with 1+ mental health condition visit, in any of the 3 services (inpatient, outpatient, ED), at each of the sites, in the prior 12 months. The numerator was the count of distinct patients in with a MH visit in each of the 3 services.

Additional File 15, Figure 7. *Annual rolling percentages of patients <21 years-old with an identified mental health condition/all patients, by Service (Inpatient, outpatient, ED), and diagnostic category, 2010 to 2023.*
Annual rolling proportions were computed each month using denominator that were the count of all patients (not necessarily MH) in each of the 3 services (1+ visit in inpatient, outpatient, ED) in the prior 12 months and the 16 condition categories. The numerator was the count of distinct patients with at least one mental health condition during this same time-period.

Additional File 16, Figure 8. *Annual rolling percentages of patients <21 years-old with an identified mental health condition by Service (Inpatient, outpatient, ED), and diagnostic category, 2010 to 2023.*
Annual rolling proportions were computed each month using denominator that were the count of all patients with 1+ mental health condition visit of that type, in each of the 3 services (inpatient, outpatient, ED) in the prior 12 months and the 16 condition categories. The numerator was the count of distinct patients with a MH condition in that particular category, in each of the particular services.

Additional File 9, Figure 1. Annual rolling percentages of patients <21 years-old with an identified mental health condition/all patients, by site, 2010 to 2023.

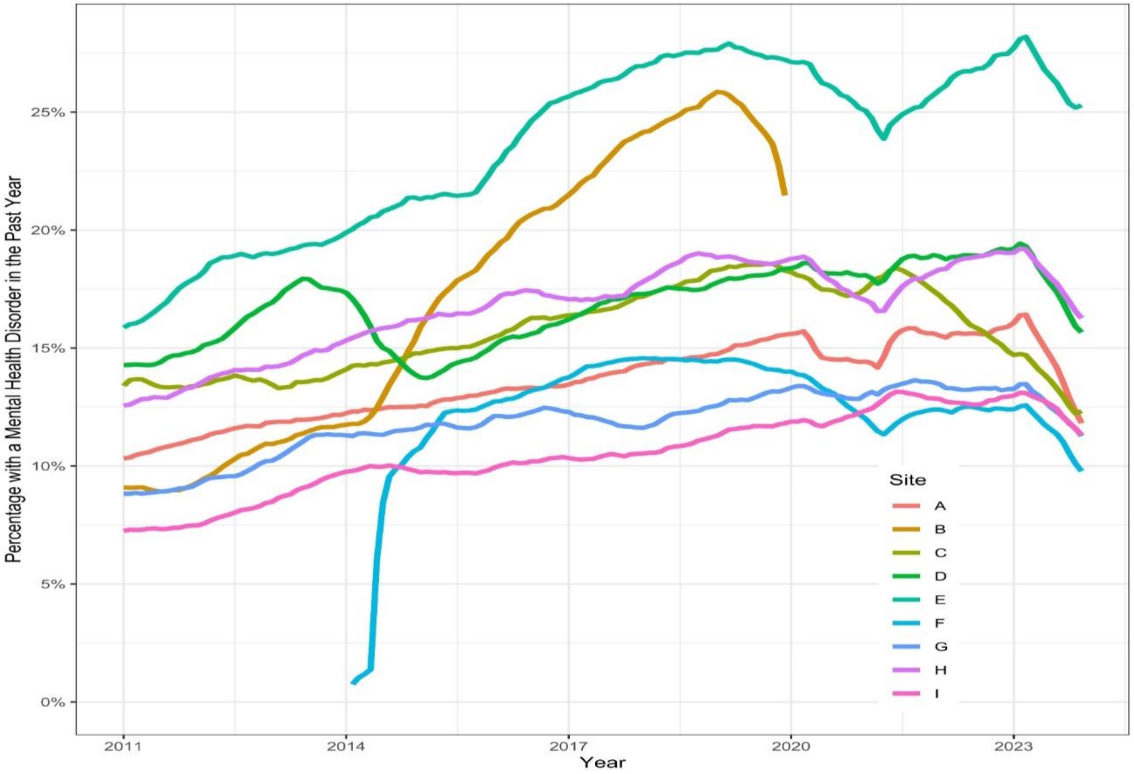

Count of distinct patients with 1+ MH disorder /
Count of patients in each of the 9 sites

Over the 13-years of the study, the total percentage of patients with a MH disorder rose from 10.6% in 2010 to 15.1% in 2023, with some variations among the 9 participating sites.

Trajectories over time show increasing rates, peaking up to 15.4% prior to Covid-19 pandemic. At the beginning of 2020, decreasing rates are noted at all sites, with reversals in 2021, with continued increases or plateauing at most sites except for one (Site C) were rates decreased.

Prior to the pandemic, the increased rates are likely attributed to expansion of mental health services within the pediatric setting as well as increased awareness and MH screenings in pediatric settings. Site D trajectory decrease in rates in 2014-2015 coincide with an expansion of pediatric hospital beds (increasing denominator).

Additional File 10, Figure 2. Annual rolling percentages of patients <21 years-old with an identified mental health condition/all patients, by condition category and site, 2010 to 2023.

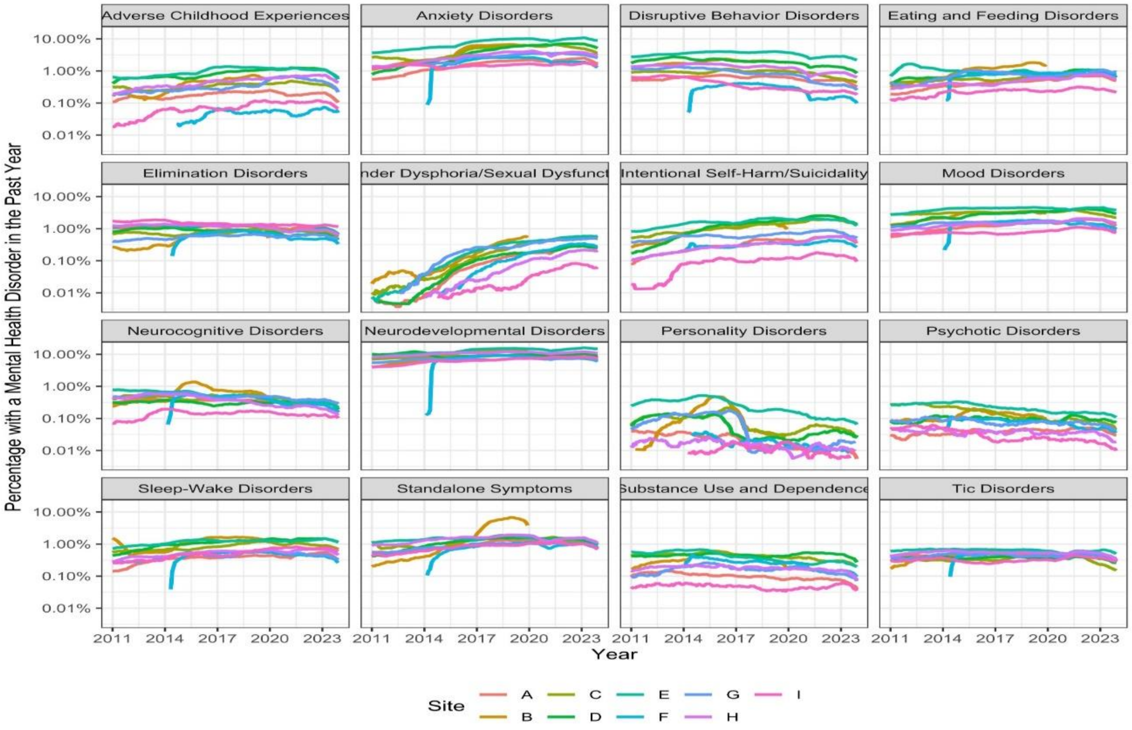

Condition categories with varying increasing rates, across the 9 sites include exposure to ACEs, anxiety disorders, eating disorders, gender dysphoria, intentional self-harm, mood disorders, neurodevelopmental disorders, sleep wake disorders and standalone symptoms.

Additional File 11, Figure 3. Annual rolling percentages of patients <21 years-old with an identified mental health condition/all patients, by Service (Inpatient, outpatient, ED), 2010 to 2023.

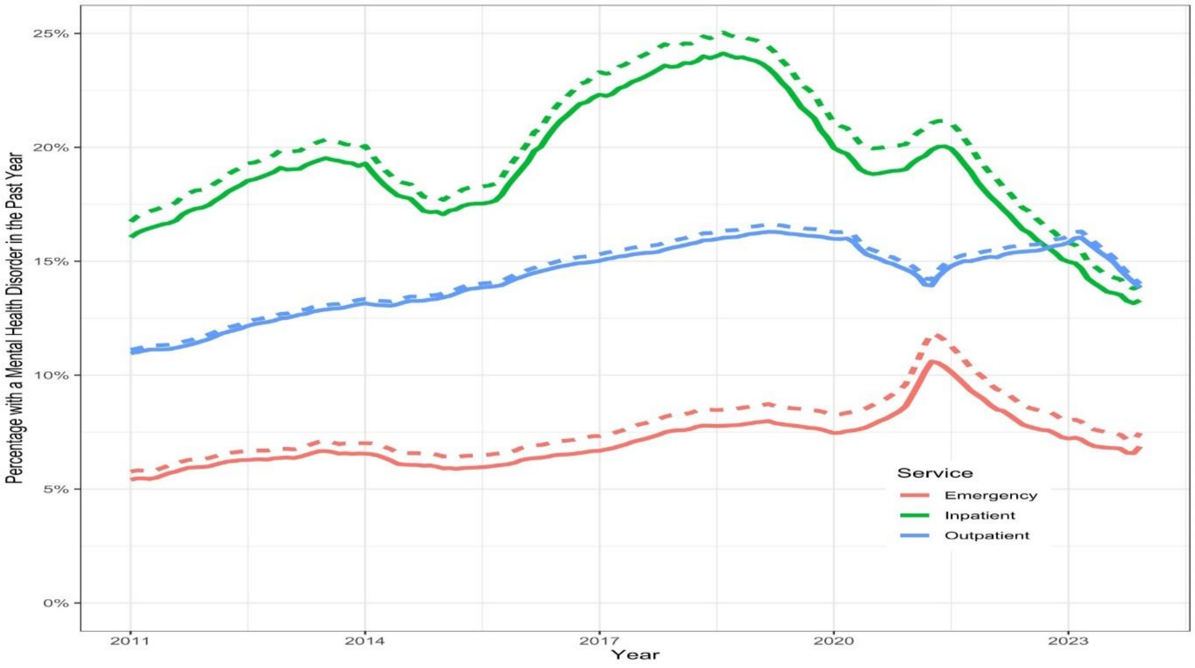

Count of distinct patients with 1+ MH condition/Count of all patients in each of the 3 services

Increasing rates of patients with 1+ MH condition are seen in each of the 3 services up to 2019, prior to Covid-19.

The trajectories show steady increases in the outpatient services. The decreases noted in the inpatient services may be due to the increases in pediatric beds (increasing the denominator).

In 2020, rates decrease sharply in inpatient and outpatient services while an even sharper increase is noted in the ED, coinciding with the Covid quarantine.

Percentages of those for ACE are slightly higher than those without Aces but follow similar trajectories.

Additional File 12, Figure 4. Annual rolling percentages of patients <21 years-old with an identified mental health condition/all patients, by Service (Inpatient, outpatient, ED), and site, 2010 to 2023.

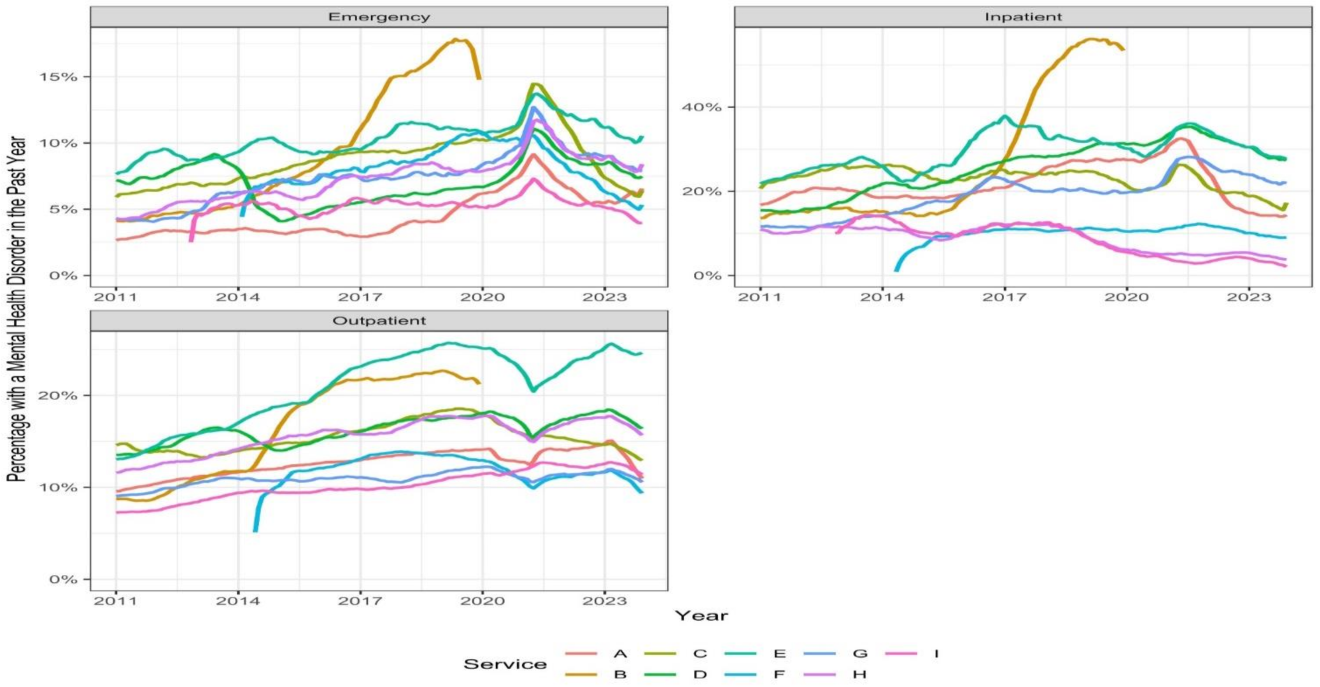

In the outpatient services, the percentage of patients with mental health conditions increased at all sites, with some annual variations, up to 2019, prior to Covid-19 pandemic.

Coinciding with the Covid Pandemic in 2020, there are sharp increases in ED visit across most sites, and sharp decreases in outpatient visits at most sites. These change direction in 2022 with decreasing ED visits and increasing outpatient sites (except at Site C where there are decreasing visits.

At Site D there was a decrease in 2014, while site B showed a significant increase in 2014, and Site E in 2016.

The inpatient service showed more variability with sharp increases at Site B and Site E in 2015-2016 and decreases at Sites F and H. The decrease in 2013-2014 at Site D may be attributed to higher admissions with the opening of a larger pediatric hospital.

With the onset of Covid-19, there were sharp decreases in outpatient services and sharp increases in ED services.

Additional File 13, Figure 5. Annual rolling percentages of patients <21 years-old with an identified mental health condition by Service (Inpatient, outpatient, ED), 2010 to 2023.

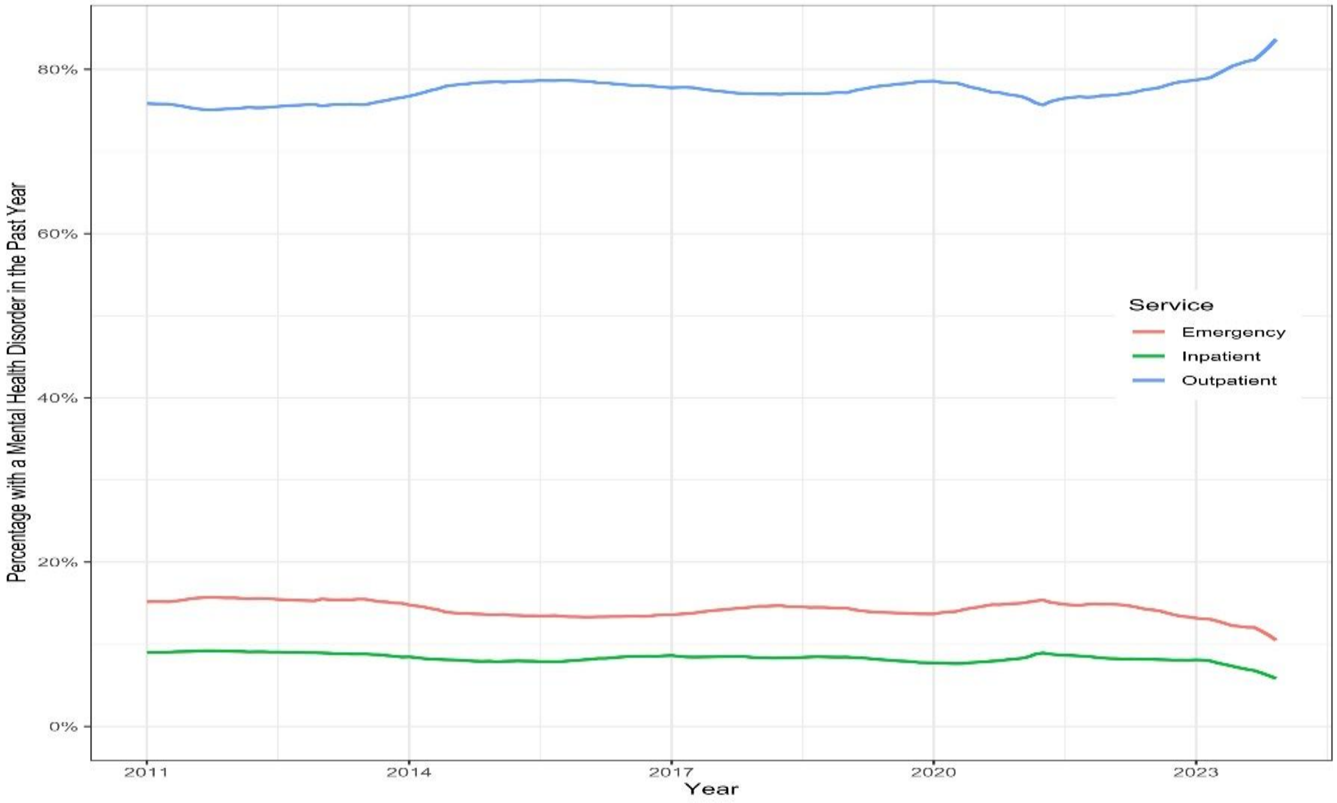

The count of distinct patients in each of the 3 services with MH conditions/ Count of all distinct patients with 1+ MH conditions in any of the 3 services

The outpatient services are the highest utilized services by the MH patients, the ED showing much lower rates and inpatient pediatric service, having the lowest rates.

While the rates of MH disorders increased throughout the study period 2012-2023, the proportion of MH patients in each of these 3 services remains roughly the same in the inpatient service, slightly increasing in outpatient and slightly decreasing in ED service.

With onset of Covid, at the beginning of 2020, a decrease is seen in the outpatient service, the ED showing an increase while the inpatient remains the same.

Additional File 14, Figure 6. Annual rolling percentages of patients <21 years-old with an identified mental health condition by Service (Inpatient, outpatient, ED), and site, 2010 to 2023

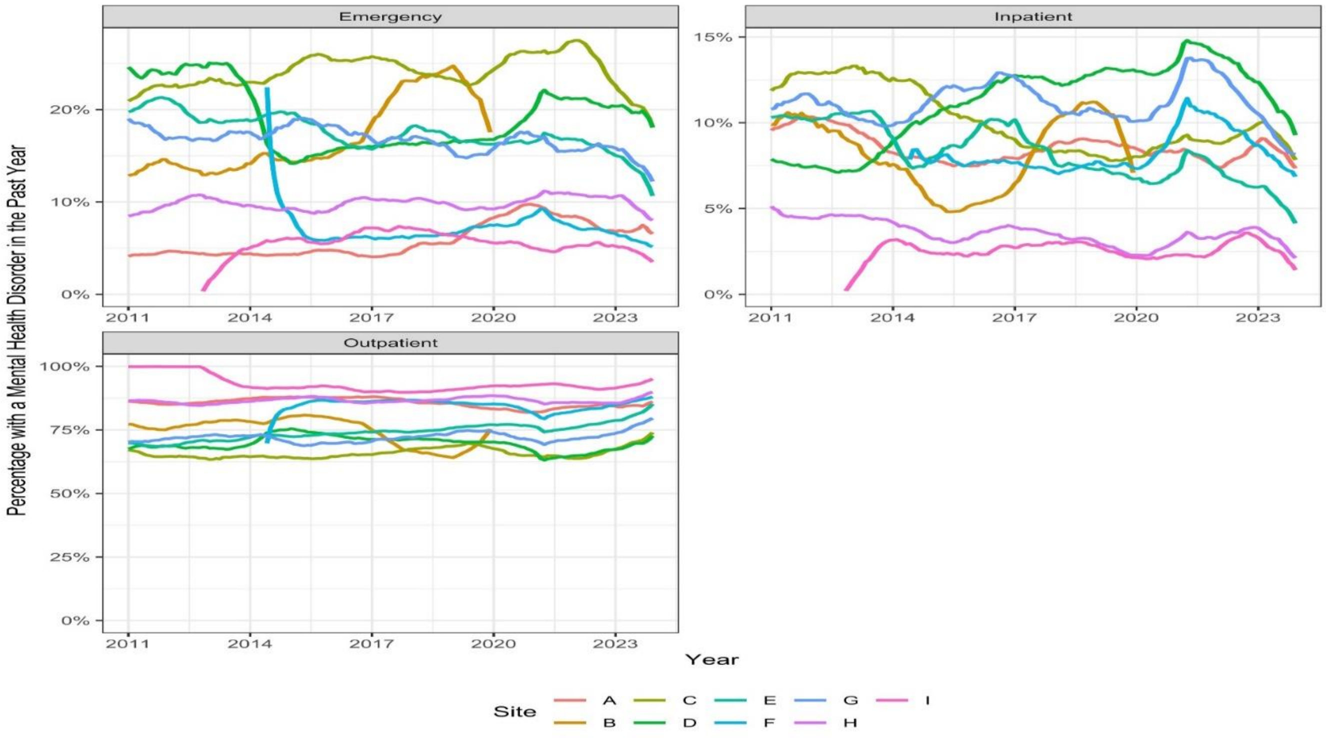

Count of distinct patients with MH conditions, in each of the 3 services/ Count of all patients with 1+ MH in any each of the 3 services at each of the sites.

The percentages of patients with a MH condition clustered below 20% for inpatient services and above 60% for outpatient services at all sites, while ED utilization varied between 5% to 30% among the nine sites.

Additional File 15, Figure 7. Annual rolling percentages of patients <21 years-old with an identified mental health condition/all patients, by Service (Inpatient, outpatient, ED), and diagnostic category, 2010 to 2023.

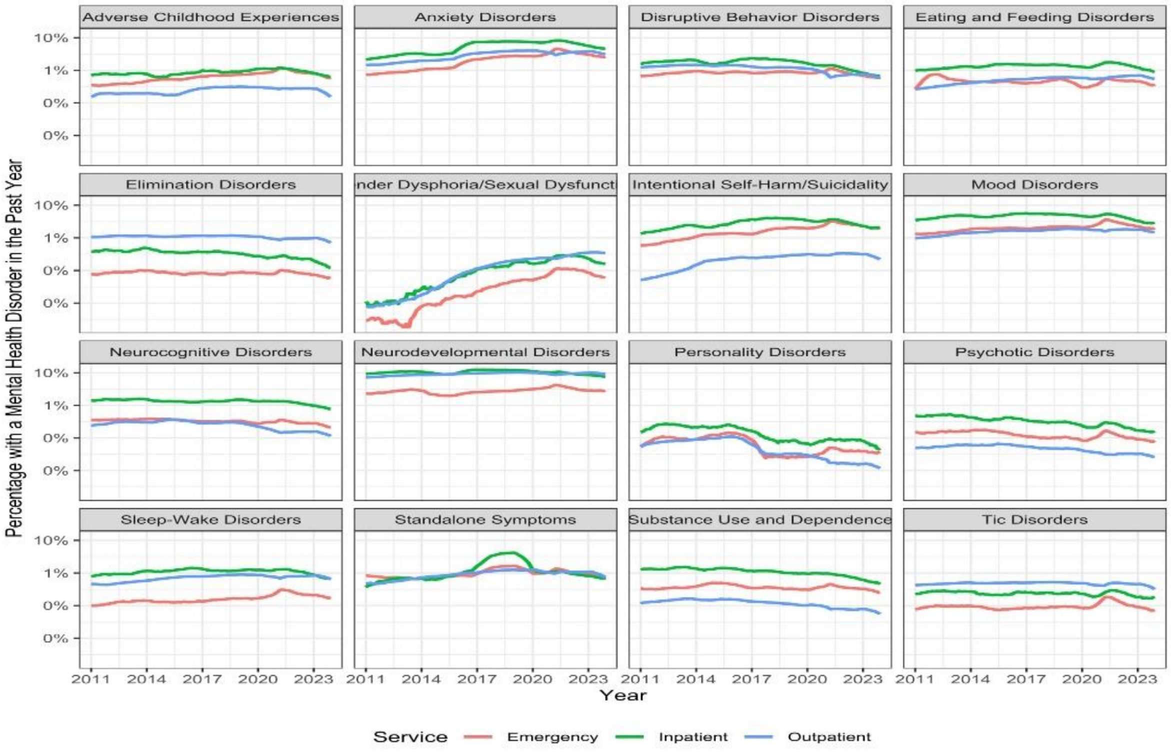

Most diagnostic categories, other than tic and elimination disorders, had higher percentages of MH patients in the outpatient vs inpatient services.

The ED service had the lowest rates for most, except for adverse childhood experience, intentional self-harm, psychotic disorders, and substance use.

Throughout the study period, increasing estimates were noted for anxiety disorders, gender dysphoria, intentional self-harm, sleep-wake disorders across the 3 services.

Sharp increases were noted adverse childhood experience category and intentional self-harm in ED services.

Variations coinciding with the Covid-19 pandemic are noted for most the diagnostic categories and services. The most prominent being the sharp increases in ED services at the same that outpatient services decreased or stayed the same.

Additional File 16, Figure 8. Annual rolling percentages of patients <21 years-old with an identified diagnosed mental health condition by Service (Inpatient, outpatient, ED), and diagnostic category, 2010 to 2023.

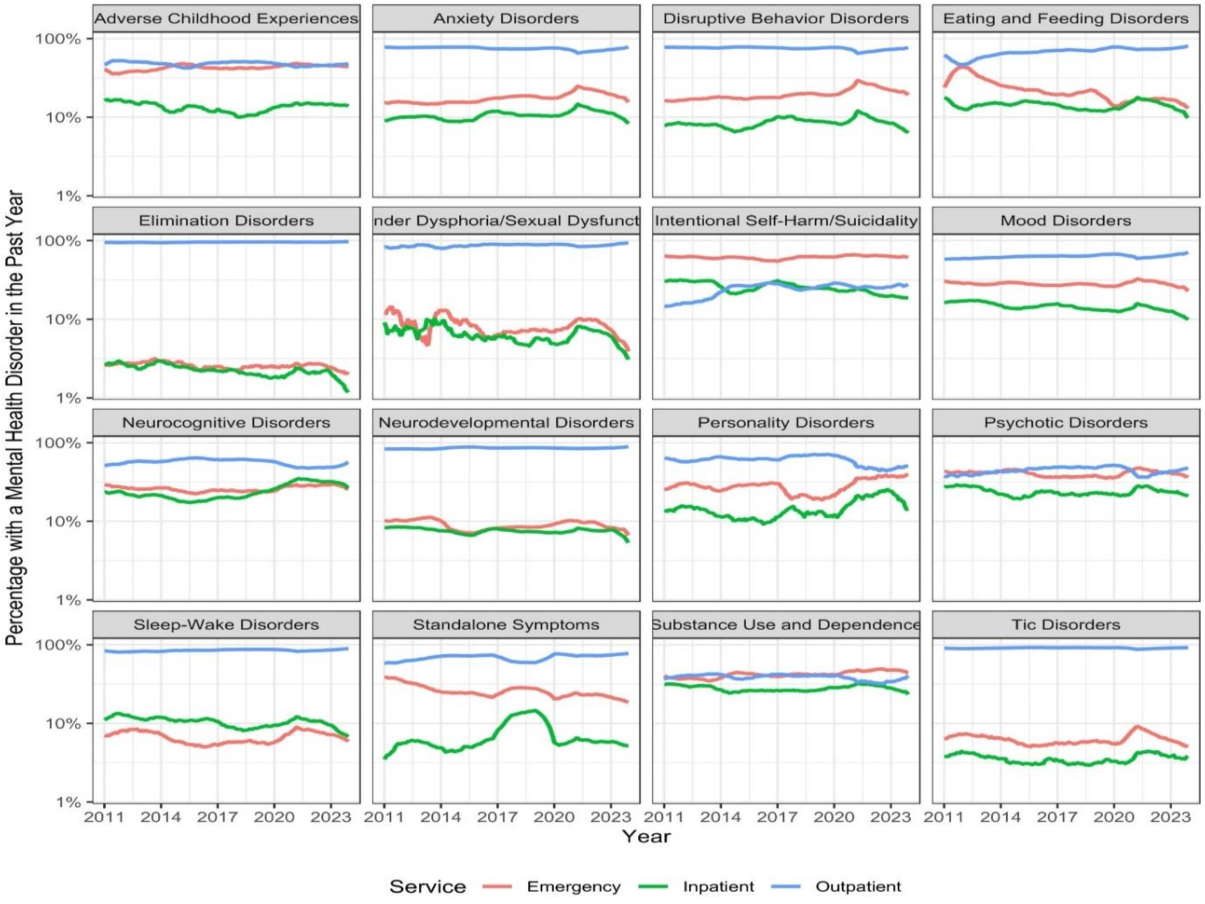

Count of distinct patients with MH condition in each of the 16 categories and 3 services/ Count of all patients with 1+ MH condition in each of the 3 services.

The percentage of patients with MH conditions compared to all patients were lowest in the inpatient setting, for all diagnostic categories and highest for outpatient services in most categories. The percentage with intentional self-harm/suicidiality was highest in ED setting.

Adverse childhood experience, psychotic and substance use disorders clustered around similar rates in outpatient and ED.
